# The Evolving Scenario of ES-SCLC Management: From Biology to New Cancer Therapeutics

**DOI:** 10.3390/genes15060701

**Published:** 2024-05-27

**Authors:** Pamela Trillo Aliaga, Ester Del Signore, Valeria Fuorivia, Gianluca Spitaleri, Riccardo Asnaghi, Ilaria Attili, Carla Corvaja, Ambra Carnevale Schianca, Antonio Passaro, Filippo de Marinis

**Affiliations:** 1 Division of Thoracic Oncology, IEO, European Institute of Oncology IRCCS, Via Ripamonti 435, 20141 Milan, Italy; 2 Division of New Drugs and Early Drug Development for Innovative Therapies, European Institute of Oncology IRCCS, 20141 Milan, Italy; 3 Department of Oncology and Haematology (DIPO), University of Milan, 20122 Milan, Italy

**Keywords:** SCLC, immunotherapy, atezolizumab, durvalumab, RB, TP53, SCLC subtypes, PARP inhibitors, BITEs, tarlatamab

## Abstract

Small cell lung cancer (SCLC) is an aggressive neuroendocrine carcinoma accounting for 15% of lung cancers with dismal survival outcomes. Minimal changes in therapy and prognosis have occurred in SCLC for the past four decades. Recent progress in the treatment of extensive-stage disease (ES-SCLC) has been marked by incorporating immune checkpoint inhibitors (ICIs) into platinum-based chemotherapy, leading to modest improvements. Moreover, few second-line-and-beyond treatment options are currently available. The main limitation for the molecular study of SCLC has been the scarcity of samples, because only very early diseases are treated with surgery and biopsies are not performed when the disease progresses. Despite all these difficulties, in recent years we have come to understand that SCLC is not a homogeneous disease. At the molecular level, in addition to the universal loss of retinoblastoma (RB) and TP53 genes, a recent large molecular study has identified other mutations that could serve as targets for therapy development or patient selection. In recent years, there has also been the identification of new genetic subtypes which have shown us how intertumor heterogeneity exists. Moreover, SCLC can also develop intratumoral heterogeneity linked mainly to the concept of cellular plasticity, mostly due to the development of resistance to therapies. The aim of this review is to quickly present the current standard of care of ES-SCLC, to focus on the molecular landscapes and subtypes of SCLC, subsequently present the most promising therapeutic strategies under investigation, and finally recap the future directions of ongoing clinical trials for this aggressive disease which still remains a challenge.

## 1. Introduction

Small cell lung cancer (SCLC) accounts for approximately 10–15% of all lung cancers. It presents clinically aggressive malignant behavior and is characterized by rapid tumor growth, high vascularity, and early metastatic dissemination [1]. Approximately two-thirds of patients with SCLC are diagnosed with extensive-stage disease (ES-SCLC) [2]. Annually, it is estimated that SCLC kills 200,000 to 250,000 patients worldwide [3].

The main risk factor for SCLC is smoking, as more than 95% of patients with SCLC are current or former smokers. Only a small percentage of SCLC cases occur in patients without a history of smoking, and these patients have different outcomes and genomic profiles [4,5,6].

SCLC is characterized by rapid responses to chemotherapy (CT) and sensitivity to radiotherapy [1]. For about 40 years the standard first-line treatment for ES-SCLC consisted of platinum-etoposide (PT/EP) therapy [7]; however, despite the initial higher response rates (RR) (60–78%) most patients relapsed within 6 months and the median overall survival (mOS) was about 10 months [8]. Currently, the first-line standard of care consists in chemo-immunotherapy, which adds only a modest survival benefit [9,10]. Furthermore, there are only a few treatment options available in the latest lines [2,11].

Disease progression in SCLC is characterized by a rapid transition from the initial chemoresponsive state to the subsequent chemoresistant state. The mechanisms responsible for this acquired therapeutic resistance have not been defined. Some studies have proposed epithelial-to-mesenchymal transition (EMT) as a potential mechanism of resistance [12,13]. However, this topic remains under investigation.

SCLC is approached as a single disease entity because it has been traditionally considered a homogeneous disease both from clinical and pathological point of view [14]. Clinical trials for SCLC have largely focused on unselected populations and have produced disappointing results. A better understanding of the molecular characteristics of SCLC is necessary to personalize therapeutic strategies. So, the characterization of this single entity needs to be refined to understand and manage the inter- and intratumoral heterogeneity [15]. In fact, it is emerging that SCLC is not a single disease but that there are molecular subtypes.

The aim of this review is to quickly present the current standard of care of ES-SCLC, to focus on the molecular landscapes and subtypes of SCLC, subsequently present the most promising therapeutic strategies under investigation, and finally recap the future directions of ongoing clinical trials.

## 2. Current Treatment Options

### 2.1. First Line Treatment

Since the 1980s, the first-line treatment for patients with ES-SCLC has been PT/EP combination [7], which showed a benefit in terms of survival compared to other treatments [16,17]. After about 40 years, this standard was renewed by adding the immune checkpoint inhibitor (ICI) to CT [9,10].

Atezolizumab (an anti-programmed cell death protein 1 ligand, PD-L1) was the first ICI approved in combination with carboplatin (CP) plus EP according to the results of IMpower-133, a double-blind, placebo-controlled, phase III trial in 403 untreated patients randomized to receive 1:1 CP/EP plus atezolizumab or CP/EP plus placebo for four cycles in the induction phase, followed by maintenance with atezolizumab or placebo until progression or unacceptable toxicity. The mOS was 12.3 months in the atezolizumab arm versus (vs.) 10.3 months in the placebo arm (hazard ratio [HR] 0.70; 95% confidence interval [CI], 0.54 to 0.91; *p* = 0.007) and the median progression-free-survival (mPFS) was 5.2 months in atezolizumab arm vs. 4.3 months in placebo arm (HR 0.77; 95% CI: 0.62 to 0.96; *p* = 0.02), with an acceptable safety profile (grade 3 or 4 adverse events [AEs] occurred in 56.6% patients in the experimental arm and in 56.1% in CT arm; immune-related adverse events [irAEs] occurred in 39.9% vs. 24.5%, respectively) [9]. The efficacy and safety of this combination were confirmed in the subsequent update of the study [18,19]. IMbrella A is a phase IV, single arm, long-term observational study in 18 patients who were still receiving atezolizumab, or had discontinued the drug and were in survival follow-up at the time of IMpower133 closure, with a 5-year OS rate of 12% [20].

The second ICI approved in combination with PT/EP was durvalumab (another anti-PD-L1) according to the CASPIAN trial [10], an open-label phase III trial in 805 untreated patients randomized to receive 1:1:1 durvalumab plus PT/EP, durvalumab in combination with tremelimumab (an anti-cytotoxic T-lymphocyte-associated antigen 4, CTL4) plus PT/EP, or PT/EP alone until four cycles in the combination arms or six cycles in the CT arm, followed by maintenance with durvalumab or durvalumab plus tremelimumab in the experimental arms. The combination arm with durvalumab showed a significant benefit in terms of mOS (13.0 months in the CT/ICI arm vs. 10.3 months in the CT arm; HR 0.73; 95% CI: 0.59–0.91; *p* = 0.0047) and a good safety profile (grade 3 or 4 AEs occurred in 62% in the experimental arm and 62% in chemotherapy arm; irAEs were 20% vs. 3%, respectively) [10]. The 3-year OS update confirmed the preliminary data in terms of efficacy (36-month OS rate was 17.6% in the experimental arm vs. 5.8% in the CT arm; HR 0.71; 95% CI: 0.60–0.86; *p* = 0.0003) and safety [21]. On the other hand, the combination of durvalumab with tremelimumab plus CT was not associated with a significant improvement in mOS compared to the CT arm (10.4 months vs. 10.5 months, respectively; HR 0.82; 95% CI: 0.68–1.00; *p* = 0.045), with a worse toxicity profile than the durvalumab arm (serious AEs [SAEs] occurred in 45% in the durvalumab plus tremelimumab plus CT arm, vs. 32% in the durvalumab plus CT arm, vs. 36% in the CT arm) [21,22].

At the same time as the IMpower-133 and CASPIAN trials, other ICIs were evaluated in combination with PT/EP combination in the first-line setting of ES-SCLC (Table 1). In the phase II trial ECOG-ACRIN EA5161, nivolumab (an anti-PD-1) significantly improved PFS and OS when combined with CT [23]. In the KEYNOTE-604 trial, pembrolizumab (an anti-PD-1) numerically improved PFS and OS; however, OS did not reach statistical significance [24]. Subsequently, several trials largely conducted in selected countries (mainly in China) have confirmed that adding an ICI (the anti-PD-L1 adebrelimab in the CAPSTONE-1 trial, or the anti-PD-1s serplulimab, toripalimab, and tislelizumab in the ASTRUM-005, EXTENTORCH, and RATIONALE-312 trials, respectively) to CT improved both mPFS and mOS (HR ranging from 0.63 to 0.79) [25,26,27,28].

The addition of an anti-CTLA4 (ipilimumab) to PT/EP did not improve OS compared to CT alone as demonstrated in a previous trial (CA184-156), confirming the disappointing results of tremelimumab in the CASPIAN trial [21,22,29].

Recently, several retrospective studies and phase IIIb trials were conducted in different countries to evaluate the safety of the new standard in a real-world setting, allowing the inclusion of patients with poor Eastern Cooperative Oncology Group performance status (ECOG-PS 2) and the extension of the induction phase up to six cycles of chemotherapy in the experimental arm [30,31,32,33,34,35,36,37]. These studies demonstrated non-addictive toxicity by prolonging the induction phase up to six cycles; however, the number of patients with ECOG-PS 2 was too low to identify the benefit and tolerability of the ICI plus CT combination in this subgroup population (Supplementary Data, Table S1).

### 2.2. Maintenance Treatment

Although the addition of either anti-PD-L1 or PD-1 to upfront CT has improved prognosis and become the new standard, two clinical trials testing the addition of an ICI in the maintenance phase after induction CT were negative [38,39]. Pembrolizumab was evaluated in a small phase II trial that included 45 patients, and the results of this study showed no benefit in terms of mPFS (1.4 months; 95% CI: 1.3–2.8) and OS (mOS was 9.6 months and 1-year OS rate was 37%; 95% CI: 7.0–12) [38]. The CheckMate-451 study was a phase III randomized (1:1:1) trial that investigated the efficacy of maintenance with nivolumab or nivolumab plus ipilimumab or placebo after four cycles of CT in 834 patients. The addition of either anti-PD-1 or anti-CTLA-4 in maintenance did not improve mOS (9.2 months for nivolumab plus ipilimumab, 10.4 months for nivolumab, and 9.6 months for placebo) [39]. Considering these data, in patients treated in the first line with CT only, immunotherapy as maintenance treatment is not an option and it is strongly recommended to start with CT plus ICI already from the induction phase [2,11].

### 2.3. Second Line and Beyond

Subsequent treatment of ES-SCLC depends on the CT-free interval (CTFI). According to the European Society Medical Oncology (ESMO) guidelines, if CTFI is ≥90 days then patients are considered “sensitive” and can be re-treated with the same first-line regimen. Instead, if CTFI is <90 days then patients are considered “resistant/refractory”, receive no benefit from the same regimen, and they are poor responders to subsequent treatments with a consequent poor prognosis [2,40,41]. Instead, the National Comprehensive Cancer Network (NCCN) makes use of different cutoffs to select patients with sensitive disease: ≥6 months [11]. However, the CTFI cutoffs above were based on old data, before the advent of ICI in the first-line setting. For this reason, some recent retrospective studies discussed the possibility to review the cutoffs considering the new standard treatment [42,43].

For many years, topotecan has been considered the referral second-line for patients with relapsed SCLC on the basis of two pivotal trials of intravenous or oral topotecan compared to CAV (cyclophosphamide, adriamycin, vincristine) or best supportive care (BSC), respectively [44,45]. Its efficacy varies according to PT sensitivity, with an ORR of 37% vs. 6% and an mPFS of 4.2 months vs. 1.9 months in patients with sensitive and refractory SCLC, respectively [46]. However, topotecan was approved for resistant/refractory SCLC based on comparison phase III trials with CAV and amrubicin, where topotecan showed a better safety profile with similar mOS [44,47] (Table S2).

PT-based regimen rechallenge is an option for patients with solely CT-sensitive disease based on four “historical” phase II and retrospective studies [48,49,50,51] (Table S3). Recently, a randomized phase III trial of PT-based CT rechallenge over oral topotecan has showed that the reuse of the same regimen guarantees the best ORR (49% vs. 25%) and a longer mPFS (5.4 months vs. 4.1 months) for a similar mOS (7.5 months vs. 7.4 months) [51].

Lurbinectedin is a selective inhibitor of oncogenic transcription that prevents transcription factors from binding to their recognition sequences, leading on the one hand to tumor cell apoptosis and on other hand influencing the tumor microenvironment by inhibiting tumor-associated macrophages (TAM) [52,53]. In June 2020, the Food and Drug Administration (FDA) granted accelerated approval of lurbinectedin as a new second-line treatment for relapsed SCLC, according to the results of an open-label phase II, single-arm basket trial, where 105 patients (43% resistant and 57% sensitive) received lurbinectedin as single agent, with an ORR of 35.2% (95% CI: 26.2–45.2), mPFS of 3.5 months, mOS of 9.3 months, and a favorable safety profile in terms of hematological toxicities [48]. Lurbinectedin performed better in patients with CT-sensitive SCLC [54]. However, these promising data have not been confirmed in a randomized open-label phase III trial, ATLANTIS, where 613 patients with relapsed SCLC received lurbinectedin plus doxorubicin or standard therapy (CAV or topotecan according to the investigator’s choice), resulting in an mOS of 8.6 months vs. 7.6 months (HR 0.97; 95% CI: 0.82–1.15; *p* = 0.70) for lurbinectedin plus doxorubicin compared to the standard therapy, respectively, but with a favorable hematological safety profile compared to standard [55]. A post-hoc subgroup analysis of the two trials of lurbinectedin in elderly patients > 65 years old (147 patients) showed that in this subgroup, lurbinectedin was superior to the standard of care both in terms of efficacy and safety [56]. Despite the failure of the phase III confirmatory trial, the FDA has not rescinded approval for lurbinectedin, based on the positive data of a phase I/II trial with lurbinectedin plus irinotecan in 21 patients with relapsed SCLC [57]. Furthermore, a recent phase II trial (LUPER) of lurbinectedin in association with pembrolizumab in 28 patients with SCLC relapsing to first-line CT (without ICI) confirmed its activity with an ORR of 46% and an mPFS of 5.3 months [58] (Table S4). Ongoing trials of lurbinectedin are depicted in Table S5. The IMFORTE trial is a randomized phase III trial of maintenance of lurbinectedin in association with atezolizumab over the standard of care (atezolizumab monotherapy) in patients with SCLC whose disease is not progressing to first-line CT plus atezolizumab (NCT05091567) [59]. A new confirmatory ongoing open-label phase III trial was designed (the LAGOON study [NCT05153239]), where patients who had progressed to first-line CT/ICI were randomized (1:1:1) to receive lurbinectedin as single agent, lurbinectedin plus irinotecan, or standard therapy, according to investigator’s choice (topotecan or irinotecan) [60].

In the past few years, ICIs have been also tested in relapsed SCLC (Table S6). Pembrolizumab (KEYNOTE-028 and -158) and nivolumab ± ipilimumab (CheckMate-032) showed modest activity, with ORR ranging from 11.6% to 33% and mOS from 5.7 months to 9.7 months, while atezolizumab (IFCT-1603) failed to be superior to second-line CT [61,62,63,64,65]. Subsequently, the randomized phase III trial CheckMate-331 comparing nivolumab vs. second-line CT (topotecan or amrubicin) failed to demonstrate a survival benefit for nivolumab (mOS 7.5 months vs. 8.4 months; HR 0.86; 95% CI: 0.72–1.04; *p* = 0.11) [66]. On the basis of these negative findings taken together with those of KEYNOTE-604 (first-line pembrolizumab plus CT) and CheckMate-451 (maintenance nivolumab) [17,39], the FDA decided to withdraw the approvals of nivolumab and pembrolizumab, which were previously given in 2018 and 2019, respectively [67,68].

## 3. Biology of SCLC: Molecular Landscape

The major obstacle to the advancement of therapeutic paradigms for SCLC has been the limited availability of tumor samples for detailed molecular characterization. In part, this is because surgical resection is uncommon, thus limiting access to samples for analysis. Furthermore, available tumor biopsies are often small and necrotic, and rebiopsy at the time of disease progression is not routinely performed.

To date, four major comprehensive genomic analyses of SCLC have shed light on the principal molecular pathways in SCLC development [69,70,71,72]. In 2012, double p53 and RB1 (retinoblastoma 1) loss was discovered in human SCLC and mouse models [69,70]. In 2015, comprehensive genomic profiles of 110 SCLCs from resected tumors were performed detecting, besides p53/RB1 loss, inactivating mutations in NOTCH family genes in around 25%, and rare kinase gene mutations [71]. In 2023, a real-world data study comprising 3600 cases of SCLC showed a more complex mutational landscape. In this study, the authors identified 91.6% and 73.5% alterations in TP53 and RB1, respectively. They identified unexpected TP53- and/or RB1-negative SCLCs and unusual STK11 (serine/threonine kinase 11) mutations. Moreover, they identified an increased mutational rate in PTEN (phosphatase and tensin homolog) of 9.9%; PI3KCA (phosphatidylinositol-4,5-bisphosphate 3-kinase catalytic subunit alpha) 5.6%; EGFR (epidermal growth factor receptor) 3.4%; KRAS (Kirsten rat sarcoma virus gene) 3.3%; NF1 (neurofibromatosis type 1) 3.3%, and KEAP1 (Kelch-like ECH-associated protein 1) 3.0%, compared to previous datasets. So, they suggest a new molecular classification of SCLC including three different molecular subgroups: RB1 and/or TP53 wild-type SCLCs, STK11 mutated SCLCs, and finally those SCLCs derived from transformation of NSCLC (non-small cell lung cancer) characterized by peculiar driven mutations such as EGFR [72].

SCLC shows an extremely high mutation rate and is likely linked to tobacco carcinogens, reflected by an elevated rate of C:G>A:T transversions compared to the neutral mutation rate observed in evolution [69]. Never-smoker patients with SCLC are reported to have different molecular alterations, the most common being EGFR, MET (mesenchymal epithelial transition), and SMD2 (mothers against decapentaplegic homolog 2) [4,5,6]. However, unlike NSCLC, these genetic alterations can hardly be identified as druggable targets, since such an SCLC transformation represents one of the mechanisms of resistance to tyrosine kinase inhibitors (TKIs) in NSCLC [73].

In this review, we describe key molecular alterations that could serve as molecular targets in the development of new drug molecules. As we will see below, many of these are already in development.

**Figure 1:**
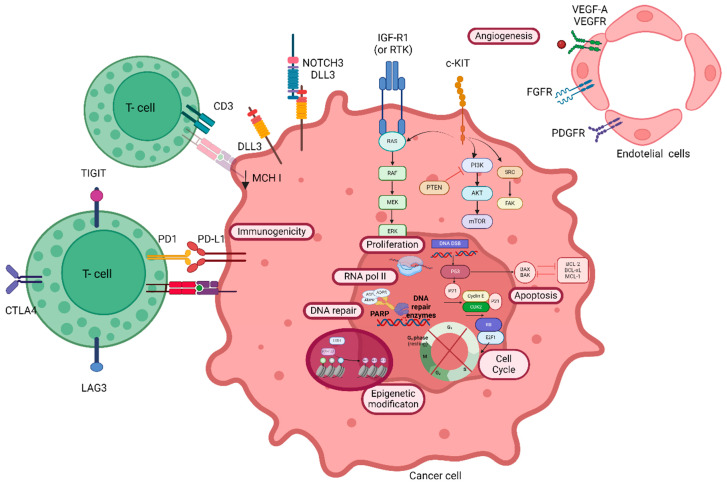
Summary of the molecular pathways potentially approachable for the development of new therapies. Legend/abbreviations: T Cell = lymphocyte T cell; PD-1 = programmed cell death protein 1; PD-L1 = PD-1 ligand; TIGIT = T-cell immunoreceptor with immunoglobulin and immunoreceptor tyrosine-based inhibitory motif [ITIM] domains; LAG3 = lymphocyte activation gene 3; DLL3 = delta-like ligand 3; NOTCH3 = neurogenic locus notch homolog receptor 3; MHC I = major histocompatibility complex class I; IGF-R1 = insulin growth factor receptor 1; RTK = receptor tyrosine kinase; VEGFR = vascular endothelial growth factor receptor; VEGF-A = vascular endothelial growth factor A; FGFR = fibroblast growth factor receptor; RAS = rat sarcoma viral gene; RAF = murine sarcoma viral oncogene; MEK = mitogen-activated protein kinase; ERK = extracellular signal-regulated kinase; PI3K = phosphatidylinositol 3-kinase; AKT = Ak strain transforming; mTOR = mammalian target of rapamycin; SRC = cellular homolog of Rous sarcoma virus; FAK = focal adhesion kinase; BCL-2/xL = B-cell leukemia/lymphoma 2 protein 2/xL; MCL1 = induced myeloid leukemia cell differentiation protein; BAX = Bcl-2 associated X-protein; BAK = Bcl-2 antagonist killer 1; RNA pol II = ribonucleic acid polymerase II; PARP = poly-ADP ribose polymerase; DNA DSB = deoxyribonucleic acid double-strand breakage; CDK2 = cyclin D kinase -2; RB1 = retinoblastoma 1; E2F1 = E2 promoter binding factor 1; ADPR = adenosine diphospho-ribosylserine hydrolase gene; LSD1 = lysine-specific demethylase 1. Created with BioRender.com (accessed on 8 April 2024).

Tumor suppressor genes have great importance in the tumorigenesis of SCLC, and loss of TP53 and RB1 genes has been found in 75–100% and 65–93% of SCLCs, respectively [69,70,71,72]. Loss of TP53 and RB1 disrupts the G1-S cell-cycle checkpoint (Figure 1). However, neither p53 nor RB1 are currently directly targetable, except indirectly using DNA-damaging agents.

The MYC gene family (c-MYC, N-MYC and L-MYC) encodes for transcription factors regulating cellular proliferation, differentiation, and apoptosis. MYC acts as oncogene and its activation and amplification has been reported in 18–31% of SCLCs and is correlated with worse survival [74]. Similarly, WEE1 is a tyrosine kinase that regulates cell-cycle progression (G2/M checkpoint regulator) primarily through phosphorylation and inhibition of cyclin-dependent kinase 1 (CDK1). Furthermore, Aurora kinases (AK-A and AK-B) are serine/threonine kinases essential for the onset and progression of mitosis (G1/M and M) and are a potential target [75,76].

Proteomic and transcriptomic analysis on SCLC samples has revealed significantly increased levels of DNA damage response (DDR) proteins, including poly-ADP-ribose polymerase (PARP) [77], checkpoint kinase 1 (CHK1), ataxia telangiectasia-mutated (ATM), and ataxia telangiectasia mutated and Rad3-related (ATR) [78]. Studies in the past few years have demonstrated the potential of targeting the DDR pathway as a promising therapeutic strategy for SCLC [79]. Similarly, Schlafen family member 11 (SLFN11) is an inhibitor of DNA replication that promotes cell death in response to DNA damage and acts as a guardian of the genome by killing cells with defective DNA replication, and about 51% of SCLCs express SLFN11 [80]. SLFN11 seems to be a predictor of response to DNA-interfering agents such as topoisomerase I and II inhibitors, platinum, and PARP inhibitors (PARPis) [81].

Regarding epigenetic regulators, both histone acetylase-modifying and DNA methylation enzymes have been described in SCLC. CREB binding protein (CREBBP) is a histone-modifying enzyme that is inactivated in about 18% of SCLCs [69,71]. However, zeste homolog 2 (EZH-2), a histone methyltrasefrase found in 62.5% of SCLCs [82], increases the expression of major histocompatibility complex class I (MHC-I) in SCLC and may represent a promising approach to improving ICI therapy [83,84]. Alternatively, lysine-specific demethylase 1 (LSD1) regulates gene expression, activates NOTCH (neurogenic locus notch homolog), and suppresses neuroendocrine features of SCLC, and represents another promising approach to enhancing the response to PD(L)-1 inhibition in SCLC [85].

Other well-known cellular pathways related to carcinogenesis are represented by receptor tyrosine kinase (RTK) and in SCLC are found alterations in IGF-1R (insulin-like growth factor receptor 1), FGFR1 (fibroblast growth factor receptor 1), KIT, MET (mesenchymal epithelial transition) and EGFR [69,70,72]. Protein levels of IGF-1 and its receptor IGF-1R are elevated in over 95% of SCLCs [86]. c-MET and its ligand HGF (hepatocyte growth factor) are functional in SCLC and are related to a worse disease prognosis [87]. Expression of c-KIT and its ligand SCF have been demonstrated in 57–88% of SCLC cell lines [88]. c-KIT activates the JAK (Janus kinase)/STAT (signal transducer and activator of transcription), PI3K, and MAPK (mitogen-activated protein kinase) pathways. In this regard, the most common intracellular pathways related to SCLC carcinogenesis are represented by the PI3K/AKT/mTOR (mammalian target of rapamycin) (activating mutations) and PTEN (loss of function) pathways [72,89,90,91,92,93].

The Hedgehog, Notch, and Wnt pathways regulate stem cell self-renewal. If abnormally activated, they can lead to neoplastic proliferation, representing an early event in tumorigenesis [94]. Notch is a tumor suppressor; it is altered in about 25% of SCLCs and represents a master regulator of neuroendocrine differentiation [71]. A well-known inhibitory Notch ligand is delta-like ligand 3 (DLL3), which is highly expressed in SCLC and minimally expressed in normal tissue, and is emerging as a promising molecular target for novel targeted drugs [95,96].

Bcl-2 (B-cell leukemia/lymphoma 2 protein) is a member of a protein family that regulates cell death (apoptosis, necrosis, and autophagy). It is an oncogene involved in the onset of SCLC and its upregulation has been found in 75–95% of SCLCs [97].

Angiogenesis plays a fundamental role in tumor growth and metastasis [98]. Studies have demonstrated that the amplification/overexpression of vascular endothelial growth factor (VEGF 1,2,4) and platelet-derived growth factor receptor-α (PDGFRα) are present in SCLC [69,72]. Thus, targeting the angiogenesis pathway is a strategy that has been actively explored in SCLC [99].

SCLCs have a relatively high tumor mutational burden (TMB) [69,72]; however, they have relatively low immunogenicity, perhaps related to low PD-L1 expression (only less than 20%) [100] and the majority (99%) are microsatellite stable (MSS) [72]. In addition, MHC class I expression is frequently downregulated in patients with SCLC [83]. However, increasing immunogenicity has become a therapeutic target to improve survival of patients with this disease.

## 4. Molecular Subtypes

All SCLC tumors were previously thought to be neuroendocrine (NE). Classic and variant subtypes of SCLC have already been defined in the past [101,102]. Distinct morphologies of these two subtypes are driven by the differential expression of two major NE transcription factors: achaete-scute homolog 1 (ASCL1) and neurogenic differentiation factor (NEUROD1). Next, the discovery of non-NE subtypes led to a re-evaluation of the SCLC classification [103,104,105]. Research has identified, in addition to ASCL1 and NEUROD1 [104,106], a third transcription factor, POU class 2 homeobox (POU2F3), which has been identified in the non-NE ‘tuft cell variant’ SCLC [107].

Zang et al. subdivided SCLC into two groups using a 50-gene expression-based NE score. Classic SCLC has medium-to-high NE differentiation, in contrast to variant SCLC which has low NE differentiation and low expression of ASCL1, but with activation of the NOTCH pathway [108,109]. Next, the consensus definition of SCLC subtypes has evolved from classic/variant to NE/non-NE with the predominance of NE-subtype (70%) to non-NE subtype (30%). Moreover, non-NE SCLC was originally described as a single subtype; however, molecular characterization has recently defined at least two non-NE SCLC subtypes [110,111].

In fact, recent studies have proposed that SCLC can be subclassified into four molecular subtypes based on the expression of lineage-specific transcription and co-transcription factors [110,111,112,113].

Rudin et al. first divided SCLC into four subtypes: SCLC-A, SCLC-N, SCLC-P, and SCLC-Y according to the relative expression of four key transcriptional regulators which include ASCL1, NEUROD1, POU2F3, and YAP1 (yes associated protein 1) [110]. The ASCL1-positive SCLC (SCLC-A) and NEUROD1-positive SCLC (SCLC-N) subtypes belong to the high NE differentiation group, and the POU2F3-positive SLCL (SCLC-P) and YAP-positive SCLC (SCLC-Y) belong to the low NE differentiation group [110]. Owonikoko et al. found that the SCLC-Y subtype was associated with high expression of interferon-γ response genes, highest weighted score on a validated 18-gene T-cell-inflamed gene expression profile score, and high expression of human leukocyte antigens (HLAs) and T-cell receptor genes. They concluded that YAP1 expression in SCLC defined a distinct subtype with T-cell-inflamed phenotype [114]. However, Baine et al. failed to identify YAP1 as a specific IHC expression in one subtype; it was scattered across the other SCLC subtypes and they concluded that YAP1 does not exclusively define a subtype [112], and this has been confirmed in another recent study [111]. Indeed, Caeser et al. identified four clusters on 37 patient-derived xenografts (PDXs)/circulating tumor cell (CTC)-derived xenografts (CDXs) based on the presence of ASCL1, NEUROD1, and POU2F3, but not on the presence of YAP1. These clusters were ASCL1-driven, ASCL1/NEUROD1-driven, NEUROD1-driven, and POU2F3-driven, respectively [115].

Gay et al. applied non-negative matrix factorization (NMF) to published data from 81 surgically resected limited-stage (LS)-SCLCs [71] and identified four SCLC subgroups: SCLC-A; SCLC-N; SCLC-P; and a new subtype with low expression of ASLC1, NEUROD1, and POU2F3 transcription factors associated with inflammatory gene signatures called inflamed-SCLC (SCLC-I) [111]. In this dataset of LS-SCLC, the four subtypes were not equally distributed: SCLC-A 36%, SCLC-N 31%, SCLC-I 17%, and SCLC-P 16%. Furthermore, Gay et al. validated their proposed molecular subtypes to the samples of treatment-naïve patients with ES-SCLC in the IMpower133 study (n = 276) and found a different distribution of subtypes: SCLC-A 51%, SCLC-N 23%, SCLC-I 18%, and SCLC-P 7% [111]. Finally, they confirmed that this classification can be defined in the absence of a tumor microenvironment using RNAseq from 62 SCLC cell lines [15]. The SLCL-I subtype shows epithelial-to-mesenchymal transition (EMT); high expression of genes related to HLAs, interferon-γ activation and immune checkpoint molecules including CD274, which encodes for PD-L1. This subtype also has the highest total immune infiltrate including T-cells, NK cells, and macrophages. They saw that SCLC-I experienced the greatest benefit from the addition of ICI to CT; in particular they found that this subtype may have the longest OS with atezolizumab plus CT. However, they observed that some patients from each subtype (SCLC-A, SCLC-N, SCLC-P, and SCLC-I) could benefit from CT/ICI (Table S7), and these data suggest that there are subgroups within SCLC subtypes that need to be identified, as we will see later. Gay et al. also reported that subtypes have distinct vulnerabilities to PT-based CT and other agents, suggesting the opportunity for personalized treatment in SCLC (Table 2). SCLC-P cell lines were sensitive to cisplatin, to PARPis, and to antimetabolite (anti-folate) and nucleoside analogs, while SCLC-N and especially SCLC-I cell lines were refractory to cisplatin. SCLC-A cell lines had a broad spectrum of sensitivity to PT: high expression of SLFN11 in SCLC-A cell lines correlated with high sensitivity to both cisplatin and PARPi. However, the mechanism underlying the sensitivity to platinum and PARis in SCLC-P is not completely known, given that in this subtype the expression of FLN11 is poor, just as the activation of the MYC pathway is not entirely clear given that its activation in SCLC-N is associated with sensitivity to Aurora-kinase-inhibitors (AKIs) while in SCLC-P it is not. The SCLC-A models may be sensitive to BCL2 inhibitors. SCLC-I cell lines highly express Bruton’s tyrosine kinase (BTK) which may represent a target; also this subgroup is the most mesenchymal subtype and drugs with epigenetic modification may alter its EMT status.

In a small study, a multiomics analysis was conducted on 19 Chinese surgical samples and 112 samples from public data [65,112]. The authors identified four clusters: 1, 2, 3, and 4. Clusters 1, 2, and 3 corresponded to Gay’s classification SCLC-I/P, SCLC-A, and SCLC-N, respectively. In this study the SCLC-P subtype samples were all included in SCLC-I with upregulated immune-related pathways. Importantly, they identified a new cluster, SCLC-C (cluster 4), characterized by a high expression of CCSP (SCGB1A1), therefore hypothesizing its origin from Clara cells that produce this protein to maintain the airway integrity. However, the small size of the validation cohort is a limiting factor of this study [116].

Further research implemented the characterization of SCLC subtypes. Ito et al., using transcriptome data from Asian SCLC tissue samples from the GSE60052 RNA sequence dataset (n = 79) [117], examined the relationships between the four molecules and some transcription and signaling molecules and they found that SCLC-A cases were more likely to express INSM1 (insulinoma-associated protein 1), DLL3, WNT11, SOX2 (sex determining region Y [SRY]-box 2), and EZH2. SCLC-N cases showed the positive expression of INSM1 and WNT11, similar to SCLC-A, but were more likely to express MYC and IGF1R. SCLC-P cases expressed NOTCH receptors, GFI1 (growth factor-independent 1), and YAP-related molecules. In contrast to SCLC-A cases, SCLC-Y cases expressed NOTCH receptors, REST (RE1-silencing transcription factor), and YAP-related molecules [118].

Another important step forward was made by a transcriptome-based analysis conducted on 271 pretreated patient samples from IMpower133. Nabet et al. identified four new NMF (non-negative matrix factorization) clusters with general concordance with Gay’s subtypes but with some differences: NMF1 = 31.4% (85/271), NMF2 = 32.4% (88/271), NMF3 = 14.4% (39/271), and NMF4 = 21.8% (59/271). The four subsets (NMF1, NMF2, NMF3, MNF4) have distinct NE phenotypes, immune composition, benefit from immunotherapy, and distinct clinical outcomes. NMF1, NMF2 and NMF3 subsets had strong NE features while the NMF4 subset appeared non-NE [119] (Table 3).

**Figure 2:**
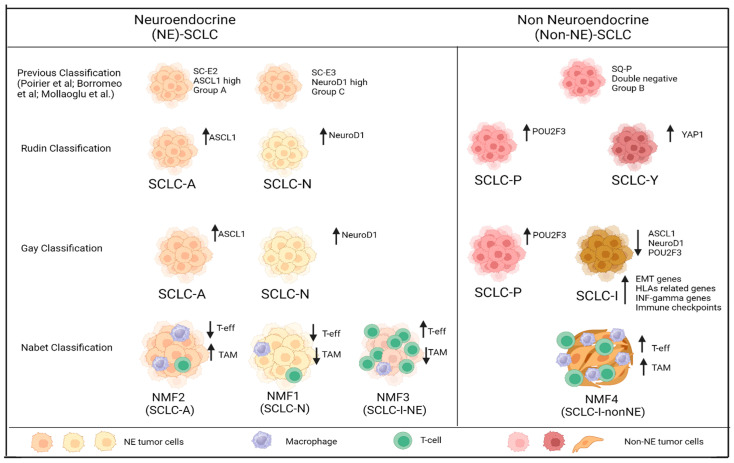
Illustrates the evolution of SCLC subtype classification over time. Legend: SC-E2, SC-E3, SQ-P = Poirier classifications according to the methylation status Abbreviations: SCLC = small cell lung cancer; NE = neuroendocrine; Non-NE = non-neuroendocrine; ASCL1 = achaete-scute homolog 1; NeuroD1 = neurogenic differentiation factor; Pou2F3 = POU class2 homeobox factor 3; YAP1 = yes-associated protein 1; EMT = Epithelial-mesenchymal transition; HLA = human leukocyte antigen; INF = inflammatory; Teff = T-effector cells; TAM = tumor-associated macrophages. Created with BioRender.com (accessed on 8 April 2024).

Importantly, they observed that ICI sensitivity is mediated by the balance between tumor-associated macrophages (TAM) and T-effector signals (Teff). They found that immune-inflamed subsets can be presented both on NE and non-NE subtypes. NE-subtype with low TAM and high Teff had major benefit from ICI/CT than non-NE that had high TAM and high Teff (NMF3 vs. NMF4). More importantly, they found that some patients who had cold-immune SCLC subtypes (SCLC-A and SCLC-n) may benefit from the addition of atezolizumab, perhaps due to the presence of T-eff high/TAM low in their samples, but the mechanism underlying this response requires further investigation [119]. In Figure 2 we illustrate the evolution of the SCLC subtype classification over time up to the most recent Nabet classification.

Subtypes and clusters describe the intertumor heterogeneity. Moreover, substantial intratumor heterogeneity has been observed in several studies [111]. In this context, although most SCLC tumor cells display strong NE features, some tumor cells do not express NE features. Emerging evidence indicates that the activation of NOTCH signaling within SCLC tumors promotes the generation of non-NE SCLC cells [108]. Qu et al. evaluated the molecular subtypes of 146 primary SCLC tumors using IHC and found that 17.6% and 2.8% of SCLC cases were positive for two and three subtype markers (ASCL1, NEUROD1, POU2F3, and YAP1), respectively [70]. Other studies demonstrated that the expression of these molecules in SCLC is not mutually exclusive, and few cases of SCLC expressed only one molecule [77,115].

The impact of intratumoral heterogeneity in the natural history of SCLC is unknown. Studies provide evidence of SCLC’s transcriptional plasticity and suggest that subtype plasticity may underlie the evolution of tumor response or resistance [68]. However, how intratumoral heterogeneity is established in SCLC tumors and contributes to the development of acquired resistance, and metastatic progression is almost unexplored. Some studies report the temporal evolution of SCLC subtypes from NE to non-NE, suggesting that these are not distinct subtypes but different stages of progressive evolution. Ireland et al. described that NOTCH promotes the dedifferentiation of tumor cells in a temporal shift from ASCL1+ to NEUROD1 to YAP+; in addition, they also suggest that MYC promotes POU2F3+ from a distinct initial tumor cell [79]. Gay et al. support subtype switching as a mechanism of acquired platinum resistance; e.g., cisplatin treatment of SCLC-A PDX induces intratumoral shifts toward SCLC-I, and this information can be used as a therapeutic strategy [68].

## 5. New Therapeutics in Development

### 5.1. Chemotherapeutic Agents

Several trials of topoisomerase-I inhibitors (irinotecan, belotecan) have been carried out in patients with SCLC (Table S8). In the second-line setting, adding irinotecan to PT/EP reuse was superior to topotecan at the cost of severe toxicity [120]. A number of phase II trials showed modest activity of irinotecan in new formulations (liposomal or hyaluronic) or in combination with an antivascular agent (apatinib) or with a proteasome inhibitor (carfilzomib) [121,122,123,124,125]. In contrast, the association of irinotecan plus dinutuximab (an anti-GD2 acting through antibody-dependent cell-mediated cytotoxicity and complement-dependent cytotoxicity) was not superior to monotherapy (topotecan or irinotecan) [126]. In the maintenance setting, a randomized phase II trial of irinotecan versus amrubicin has been prematurely closed due to a slow accrual [127]. Recently a randomized phase II trial with belotecan prolonged OS over topotecan in the second-line setting, especially in patients > 65 years old [128].

The three trials investigating the activity of alkylating agents (ifosfamide and temozolomide) in patients with relapsed SCLC showed poor activity when they were administered as monotherapy while the addition of a PARPi seemed to augment their activity, as shown in Table S8 [129,130,131].

In 2002, amrubicin, a third-generation synthetic anthracycline, was approved in Japan based on efficacy data from a phase II trial in both SCLC and NSCLC [132]. Another two phase II trials have shown a modest activity of amrubicin in a Chinese patient population [133] and with a weekly dosing schedule in a Japanese population [134]. However, this drug is not available around the world outside Japan.

Regarding other chemotherapeutic agents, clinical trials documented poor results from a novel platinum compound (loboplatin), taxanes (weekly paclitaxel or nab-paclitaxel), and antimetabolites (TAS-102) as shown in Table S8 [135,136,137,138].

### 5.2. Immunotherapy

In order to improve the effectiveness of anti-PD-(L)-1, attempts have been made to combine these ICIs with other drugs. In several studies, anti-PD1 was combined with chemotherapy (amrubicin, paclitaxel, gemcitabine, and liposomal formulation [LF]-eribulin) in pretreated patients with SCLC but with limited benefit, as reported in Table S9 [139,140,141,142]. Two phase II trial evaluated apatinib (anti-VEGFR2) plus camrelizumab (an anti-PDL-1) and anlotinib (a multi-tyrosine kinase inhibitor [mTKI]) plus penpulimab (an anti-PDL-1) [143,144]. In addition, some trials combined ICI with PARPis and these will be discussed in the dedicated chapter.

Despite the disappointing results of anti-CTLA4 drugs in the first-line and second-line settings [63,64,145], novel attempts with this class of drugs have been conducted: in the BIOLUMA phase II trial, nivolumab plus ipilimumab combination showed a remarkable ORR (38.8%) in pretreated patients at a high cost of toxicity (two treatment-related deaths), and currently the trial has been amended to enroll only patients whose disease has a high tumor mutation burden (TMB) [146]; in another randomized phase II trial, tremelimumab was added to durvalumab in combination with or without stereotactic body radiation therapy in relapsed SCLC, increasing the ORR (28% vs. 0%) but with only a slight increase in mPFS and mOS [147]. Greater expectations arise from new molecules, such as bispecific antibodies. QL1706 is a new bifunctional antibody anti-PD-1/anti-CTLA4 that has been tested in a phase II trial in combinations with CP/ET in the first-line setting (40 patients) with promising results (ORR 89.7% and mPFS 5.7 months) [148]. Cadonilimab (AK104) is another anti-PD-1/anti-CTLA4 bispecific antibody being investigated in addition to chemotherapy (NCT05901584) or in addition to chiauranib (mTKI, anti-VERGFR 1-2-3, PDGFRα, and c-KIT) (NCT05505825) in pretreated SCLC; these trials are recruiting in China and Australia.

TIGIT (T-cell immunoreceptor with immunoglobulin and immunoreceptor tyrosine-based inhibitory motif [ITIM] domains) is an ICI that is highly expressed on T cells and natural killer (NK) cells. Given that TIGIT is often co-expressed with PD-1, an anti-TIGIT molecule could theoretically amplify the antitumor effect of PD-1/PD-L1 inhibitors. However, the SKYSCRAPER-02 study, a double-blind, placebo-controlled phase III trial, showed that the addition of tiragolumab (a monoclonal antibody that binds TIGIT) to CT plus atezolizumab did not provide an additional benefit to the standard in terms of mPFS (5.4 vs. 5.6 months, HR 1.11, 95% CI: 0.89–1.38; *p* = 0.3504) and mOS (13.1 months in both groups) [149].

LAG3 (lymphocyte activation gene 3), another ICI, is overexpressed in SCLC. Preliminary data from an open-label phase II trial which enrolled 15 patients with relapsed/refractory SCLC reported promising data with clinical benefit in four patients treated with ieramilimab (anti-LAG3) and spartalizumab (anti-PD-1) (NCT03365791) [150]. IBI110 is another anti-LAG3 that is being investigated in a phase I/II study in combination with sintilimab (anti-PD-1) in patients with ES-SCLC solid tumors, and early data of this study report that IBI110 has an acceptable safety profile and promising antitumor activity (NCT05026593) [151].

Other ICIs such as immunoglobulin-like transcript 4 (ILT4) are also being explored. The KEYNOTE-B99 study is an ongoing randomized phase II trial evaluating pembrolizumab plus CP/EP in combination with either MK-4830 (an anti-ILT4 antibody), boserolimab (an anti-CD27 antibody) or lenvatinib (mTKI) in the first-line setting for ES-SCLC (NCT04924101) [152]. Other ongoing clinical trials with immunotherapy are summarized in Table S10.

### 5.3. Antiangiogenics

Studies with first-line bevacizumab (humanized monoclonal antibody directed against VEGF-A) in addition to CT followed by maintenance did not lead to improved survival [153,154,155]. However, the CeLEBrATE study is an ongoing, single-arm, phase II trial evaluating the addition of bevacizumab to the standard of care (CE/atezolizumab) [156]. The rationale for this and similar studies is that there exists a synergistic antitumor effect in the combination of PD(L)-1 and VEGF inhibition in preclinical models of SCLC [157].

Apatinib is a tyrosine kinase inhibitor of the VEGF receptor 2 (VEGFR2). Several studies have evaluated the efficacy of apatinib as monotherapy or in combination with CT and ICI [124,143,158,159,160,161,162]. The association with CT does not lead to an increase in efficacy and clinical benefit (Table S11).

The role of multitarget tyrosine kinase inhibitor (mTKI) drugs has been studied in several clinical trials [144,163,164,165,166,167,168,169] as shown in Table S12. The most encouraging results derive from ALTER 1202, a randomized Chinese phase II study which evaluated the effectiveness of anlotinib (an oral mTKI that targets VEGFR, FGFR, PDGFR, and c-KIT) vs. placebo as third or subsequent line of treatment in 120 patients with SCLC. The mPFS was significantly longer in the anlotinib group compared with the placebo group: 4.1 months vs. 0.7 months (HR 0.19, 95% CI: 0.12–0.32, *p* < 0.0001). The mOS was significantly longer with anlotinib than with placebo: 7.3 months vs. 4.9 months (HR 0.53, 95% CI: 0.34–0.81, *p* = 0.0029) [163]. In addition, anlotinib was evaluated in first-line in a placebo-controlled randomized phase III trial in patients with ES-SCLC to receive anlotinib/benmelstobart (an anti-PDL1) plus CP/EP, or anlotinib/placebo plus CP/EP, or placebo/placebo plus CP/EP. The analysis of the anlotinib plus benmelstobart plus CT over CT plus placebo confirmed the superiority of the anlotinib arm compared to CT in terms of mPFS (6.9 months vs. 4.2 months) and mOS (19.32 months vs. 11.89 months) [166,170]. However, it is important to underline some limitations of this trial. Firstly, only the Chinese population was considered, excluding other ethnic groups. Furthermore, the control arm did not involve the use of immunotherapy; rather, it exclusively involved the combination of CT + placebo, such that the obtained results are not perfectly comparable. In view of the documented clinical benefit, it is, however, necessary to conduct further studies to better evaluate this drug in clinical practice and in other countries.

It is also important to point out that the efficacy of two further multitarget drugs, pazopanib and chiauranib (both oral mTKIs that target VEGFR-1-2-3, PDGFR, and c-KIT), has been evaluated in other phase II studies with evidence of modest activity in pretreated patients [167,168,169].

Table S13 summarizes the ongoing clinical trials with first-line chemo-immunotherapy with antiangiogenics: bevacizumab, apatinib, anlotinib, lenvatinib (an oral mTKI that targets VEGFR, FGFR, PDGFR, RET, and c-KIT) and surufatinib (an mTKI against VEGFR/FGFR1/CSF1-R [colony-stimulating factor 1 receptor]). The combination of ICI and antiangiogenics is also explored in the maintenance phase (NCT05896059, NCT05509699). Moreover, there are emerging novel bispecific antibodies targeting both PD-(L)1 and VEGF, such as ivonescimab (AK112), which is combined with CP/EP in a phase Ib ongoing clinical trial in first-line ES-SCLC (NCT05116007). Other bispecific antibodies targeting PD(L)-1 and CTL4 or CD47 are explored as second-line therapy (NCT05505825, NCT05296603).

### 5.4. PARP Inhibitors

Despite promising data from preclinical models, PARPis have shown only limited activity in unselected populations of SCLC patients when used as monotherapy [171]. The combination of a PARPi with other agents has been investigated in some studies that consistently showed limited benefit from combination strategies (Table S14).

In the ECOG-ACRIN 2511 trial, the combination of veliparib compared to placebo with cisplatin and etoposide in untreated ES-SCLC led to a statistically significant improvement of mPFS (6.1 months vs. 5.5 months, HR 0.63; *p* = 0.01), without significantly improving mOS (10.3 months vs. 8.9 months, HR 0.83; 95% CI: 0.64–1.07, *p* = 0.17) [172].

The randomized phase II trial of temozolomide alone or combination with veliparib in 104 patients with relapsed SCLC, despite a documented higher ORR (39% vs. 14%) and statistically nonsignificant mOS (8.2 months vs. 7.0 months), was a negative trial since there was no significant difference in the 4-month PFS rate (the principal endpoint of the trial; 36% vs. 27%). Moreover the combination was associated with a higher incidence of G3/4 thrombocytopenia (50% vs. 9%) and neutropenia (31% vs. 7%) [130].

A different phase II trial investigated the association of olaparib with cediranib (a pan-VEGFR inhibitor) which affects sensitivity to PARPis via the downregulation of BRCA1-2 and RAD51 expression, in 25 patients with relapsed SCLC; they attained 8 PRs (28%) with a median duration of response (mDOR) of 3 months, mPFS of 4.1 months, and OS of 5.5 months, at a high cost of toxicity, with the rate of grade 3/4 AEs at 56%, the most common being hypertension, fatigue, and weight loss. Molecular analysis of homologous recombinant DNA repair genes will be performed on the baseline tumor samples [173].

In the phase II SUKES umbrella trial, patients with relapsed SCLC were enrolled and allocated based on their genomic alterations. Patients with mutations harboring homologous recombination (HR) pathway gene mutation (BRCA 1 or 2, ATM deficiency, or MRE11A mutations among the others) were allocated to the olaparib monotherapy (SUKSES-B, NCT03009682) and biomarker unselected patients were also allowed to receive olaparib and ceralasertib (an inhibitor of ataxia telangiectasia and Rad3-related [ATR] kinase) (SUKSES-N2, NCT0328607). The patient selection or the addition of an ATR inhibitor to an unselected patient population did not seem to augment the activity of the PARPi, resulting in an ORR < 10% and mPFS < 3 months in both cohorts [174].

Three trials investigated the efficacy of a PARPi and ICI combination. The first small phase II trial with 20 patients with relapsed SCLC (most with a refractory disease) treated with durvalumab plus olaparib observed four partial responses (PRs) (21.1%) at the cost of high hematological toxicity (anemia 80%, lymphopenia 60%, and leukopenia 50%) [175]; also, importantly in this trial three out four of PRs were observed in patients with inflamed phenotype [175]. A subsequent trial, a phase Ib study of fluzoparib and adebrelimab (ant-PD-L1) in 23 unselected patients with relapsed SCLC, showed poor activity [176]. Furthermore, PARPis were evaluated in the maintenance setting, and the addition of olaparib to durvalumab did not seem to prolong PFS (5.8 months) in the TRIDENT trial with respect to historical data [177].

Despite these trials demonstrating that PARPis administered alone or in association have poor activity towards an unselected patient population, Pietanza et al.’s trial demonstrated a significantly prolonged mPFS (5.7 months vs. 3.6 months) and mOS (12.2 months vs. 7.5 months) in patients with SLFN11-positive tumors treated with temozolomide plus veliparib [130]. To confirm this, the randomized phase II SWOG S1929 trial was designed to evaluate whether the addition of talazoparib to atezolizumab maintenance in patients with SLFN11-positive SCLC demonstrates a benefit in terms of higher ORR, and longer PFS and OS [178]. Indeed, it has been proven that SLFN11 inhibits HR repair by promoting the destabilization of the replication protein A-DNA complex, leading to a high sensitivity to DNA-damaging agents [179].

### 5.5. Targeted Therapy

#### 5.5.1. Inhibition of Cellular Proteins (Membrane, Plasma and Nuclear Proteins)

In this section we report studies of targeted therapies against a broad spectrum of proteins in SCLC (IGF1R, CD13R, mTOR, PLK1, MYC, AuroraKA, AuroraKB, WEE1, BCL-Xl) and summarize the results of these trials in Table S15. We observed that limited efficacy of these drugs was obtained in terms of ORR, PFS and OS [180,181,182,183,184,185,186,187,188].

The efficacy of alisertinib, an Aurora A kinase inhibitor (A-AKI), was evaluated in combination with paclitaxel vs. paclitaxel plus placebo in 178 patients diagnosed with SCLC in progression after first-line treatment in a randomized phase II trial. The mPFS was 3.32 months with alisertib/paclitaxel vs. 2.17 months with placebo/paclitaxel (HR = 0.77; 95% CI: 0.557–1.067, *p* = 0.113). In this trial, among the secondary endpoints, associations between c-MYC expression in tumor tissue (prespecified) and genetic alterations in circulating tumor DNA (retrospective) with clinical outcomes were also evaluated. Benefit on mPFS and mOS was observed among 140 patients with genetic alterations such as cyclin-dependent kinase 6 gene (CDK6), RB-like 1/2 genes (RBL1/2), and c-MYC expression (in the latter case only for mPFS) [180].

Others results come from the SUKSES trial, the first biomarker-driven umbrella study conducted in patients with recurrent SCLC [186]. This study included 286 patients with SCLC who failed platinum therapy and who had known genomic profiles based on a predesigned screening trial. Patients with MYC amplification or CDKN2A and TP53 co-alterations were allocated to adavosertib (WEE1 inhibitor) (SUKSES-C; seven patients) and those with RICTOR amplification were allocated to vistusertib (mTOR inhibitor) (SUKSES-D; four patients). Alternatively, patients who were without any predefined biomarkers were assigned to a non-biomarker-selected arm: adavosertib (SUKSES-N1; 21 patients) or AZD2811NP (a nanoparticle-encapsulated slow-release inhibitor of Aurora kinase B) (SUKSES-N3; 15 patients). However, the authors did not observe any responses either in the biomarker-selected cohorts or in the others. Despite the negative results, this trial suggests the possibility to find and use new cell-cycle inhibitor(s), according to a novel biomarker approach. Ongoing clinical trials are summarized in the second part of Table S13.

#### 5.5.2. Antibody Drug Conjugates (ADCs): Rova-T, S. Govitecan, ABBV-011 and Others

Rovalpituzumab tesirine (SC16LD6.5) (Rova-T) is an antibody–drug conjugate (ADC) directed towards DLL3. Preclinical studies in PDX tumors and early trials in patients with both limited and extended-stage SCLC showed that Rova-T induced durable tumor regression [96]. In a first-in-human phase I study, Rova-T showed encouraging results in SCLC, with an ORR of 16% in patients heavily pretreated and unselected for DLL3 expression. Importantly, in an exploratory analysis, 26 patients with high levels of DLL3 expression had an ORR of 31% [189]. TRINITY was a phase II trial of Rova-T in 339 previously pretreated (≥3L) SCLC patients with DLL3+. Around three-quarters of the patients had received two prior lines of therapy and had CT-sensitive disease, but only 17% of them had received a prior ICI. A total of 225 (66%) patients received 2 cycles of Rova-T; of these, 20 patients underwent retreatment. After a median follow-up (mFUP) of 19.1 weeks, the best ORR was 20.1% in all patients, with an mDOR of 4 months, mPFS of 3.5 months, and mOS of 5.6 months. Among patients who were DLL3-positive (≥25% of tumor cells positive for DLL3) (287 patients) and patients with high DLL3 positivity (≥75% of tumor cells positive for DLL3) (238 patients), the best ORR was 20.6% and 21.8%, respectively. The rate of grade ≥3 AEs was 54%. Pleural effusion was an event of special interest, and G3 occurred in five patients (21%) leading to three deaths [190]. The subsequent randomized (2:1) phase III trial (TAHOE study) was designed to compare Rova-T over topotecan in 417 DLL3-high SCLC patients in second line treatment. The trial was negative and the enrollment was prematurely discontinued because of no benefit on OS (principal endpoint): 6.3 months vs. 9.6 months (HR 1.46), respectively. A subgroup analysis on OS showed that both those patients with poor prognostic factors (refractory disease, stage IV at diagnosis, LDH higher than upper limit of normal, no prior PCI) and those with brain metastases were associated with shorter OS when treated with Rova-T. Despite the grade ≥3 toxicity rate being higher in the topotecan arm (88% vs. 64%, primarily hematologic toxicity), the AEs of special interest were more common in the Rova-T arm with respect to topotecan: cutaneous reaction (39% vs. 12%), edema (30% vs. 10%), pleural effusion (29% vs. 4%), pericardial effusion (20% vs. 2%), and photosensitivity reaction (16% vs. 0%) [191].

The MERU study (maintenance phase III trial of Rova-T) found no OS benefit over placebo at a preplanned interim analysis. This trial was closed in August 2019. As a result, the development of Rova-T was discontinued based on results from the TAHOE and MERU studies. AbbVie discontinued the Rova-T research and development program [192,193].

TROP2 (also known as TACSTD2) is a transmembrane receptor that can be aberrantly expressed in several cancers [194]. In a retrospective series, TROP2 was overexpressed in 18% (21/115) of high-grade NET samples; importantly, high TROP2 expression was associated with lower lung cancer-specific mortality [195]. Sacituzumab govitecan is a new ADC directed towards TROP2. Data from the phase I/II trial showed that in 50 heavily pretreated patients with SCLC who received sacituzumab govitecan, the ORR was 14%, mDOR was 5.7 months, mPFS was 3.7 months, and mOS was 7.5 months. The clinical benefit was observed both in CT-sensitive and CT-refractory SCLC patients [196]. These results were confirmed at the final results of the trial [197]. Preliminary results (mFUP of 5.1 months) from the phase II TROPiCS-03 basket trial showed that in 30 pretreated (2L) SCLC patients treated with sacituzumab govitecan, the ORR was 37% with a mDOR of 6.3 months (Table 4). The grade ≥3 AE rate was 60% and the rate of AEs leading to dose reduction was 27%. The most common AEs were diarrhea, neutropenia, constipation, fatigue, and nausea [198].

ABBV-011 is a new ADC (IG1 monoclonal antibody conjugated with calicheamicin) directed towards seizure-related homolog protein 6 (SEZ6), which is highly expressed in SCLC and high-grade NET [201,202]. In the first-in-human study, out of 445 screened patients for SEZ6, 55% met the established cutoff (≥25% tumor cells at 1+ or above); of these, 60 patients have been enrolled in the dose-expansion part of the study. Forty patients were treated at the chosen schedule of 1 mg/kg every 3 weeks. At this dose, the ORR was 25% with an mDOR of 4.2 months and mPFS of 3.5 months, respectively. Importantly they did not observe any difference in terms of ORR among patients with sensitive and refractory disease. The grade ≥3 AE rate was 65% and AEs leading to dose reduction, interruption, and discontinuation were 15%, 30%, and 8%, respectively[199].

**Figure 3:**
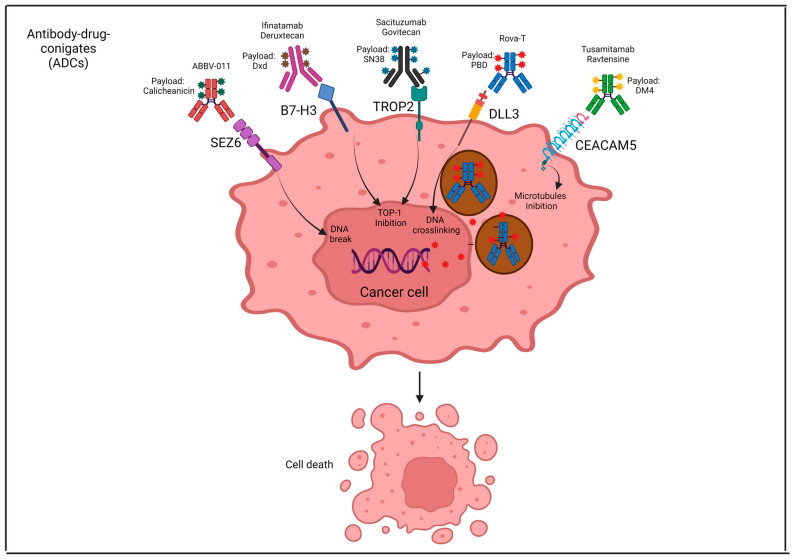
ADCs in development in SCLC. Abbreviations: ADCs = antibodies drug conjugates; SCLC = small cell lung cancer; Rova-T = rovalpituzumab tesirine; DLL3 = delta-like ligand 3; SEZ6 = seizure-related homolog protein; TROP2 = trophoblast cell surface glycoprotein antigen 2; B7-H3 = B7 homolog 3 protein; CECAM5 = carcinoembryonic antigen-related cell adhesion molecule 5; PBD = pyrrolobenzodiazepine; SN-38 = analog of camptothecin; Dxd = deruxtecan; DM4 = ravtansine; TOP1 = topoisomerase 1. Created with BioRender.com (accessed on 7 May 2024).

B7 homolog 3 (B7-H3) is a transmembrane immunoregulatory protein that is overexpressed in SCLC. Ifinatamab deruxtecan (I-DXd) is a novel B7-H3-ADC; results from the IDeate-Lung01 phase II trial were recently presented showing promising efficacy: an ORR of 52.4%; mPFS of 5.7 months, and mOS of 12.2 months [200]. Also, in the Table S16 we summarize the ongoing clinical trials with ADCs in ES-SCLC. Figure 3 depicts the ADCs in development in SCLC.

#### 5.5.3. Other Anti-DLL3 Agents: BiTE (Tarlatamab, BI-765432), TCE (HPN328) and Others

Tarlatamab (AMG 757) is a bispecific T-cell engager molecule (BiTE) which binds both DLL3 and CD3, leading to T-cell-mediated tumor lysis irrespective of MHC class I expression which is frequently downregulated in patients with SCLC [83]. DeLLphi-300 was a phase I study of tarlatamab in 141 patients with SCLC who were previously pretreated (≥1L). The maximum tolerated dose (MTD) was not reached, and the most common AEs were cytokine release syndrome (CRS) (52%) and immune effector cell-associated neurotoxicity syndrome (ICANS) (9%). Importantly, they recorded an ORR of 23.4% with an mDOR of 12.3 months irrespective of the presence/absence of baseline brain metastases [203,204]. The subsequent DeLLphi301 study was a two-dose schedule, randomized (1:1) phase II trial of tarlatamab (10 mg vs. 100 mg) in 222 patients with SCLC who were previously pretreated (≥2L). The chosen dose was 10 mg based on higher clinical benefit and better tolerance. At the dose of 10 mg, the ORR was 40% but the mDOR was not reached after an mFUP of 9.5 months; mPFS was 4.9 months and the 6-month OS was 73.4%. The AEs of special interest were CRS, which occurred in 51% (grade ≥3: 1%) and ICANS (8%) [205]. DeLLphi-305 (NCT06211036;) and DeLLphi-304 (NCT05740566) are ongoing randomized phase III trials of tarlatamab in the maintenance and second-line setting, respectively (Table 5).

BI 764532 is new analog of tarlatamab with the same mechanism of action. In the ongoing first-in-human phase I trial in 107 patients with relapsed SCLC or high-grade NE tumors (NETs), the MTD was not reached and the grade ≥3 AE rate was 27%, the most common AEs being CRS (59%), lymphocyte count decreased (20%), dysgeusia (20%), asthenia (19%), and pyrexia (18%). Among the 99 evaluable patients the ORR was 18% (25% in patients treated > 90 µg/kg) and among 39 patients with SCLC the ORR was 39% with the mDOR not reached at the date cutoff of 21 April 2023 [206].

HPN328 is a DLL3-targeting T-cell engager (TCE). This drug has three binding domains including anti-DLL3 for target engagement, anti-albumin for half-life extension, and anti-CD3 for T-cell engagement and activation. At the interim analysis, 44 patients received 0.015–24 mg of HPN328 across 11 cohorts; of these, 29 were patients with SCLC (2 patients had a PR and 1 patient had a CR). Treatment-related AEs in >10% of patients included CRS (46%), fatigue (23%), dysgeusia (21%), nausea (18%), pyrexia and vomiting (16% each), and anemia (11%). MTD determination and dose escalation are ongoing [207]. 

**Figure 4:**
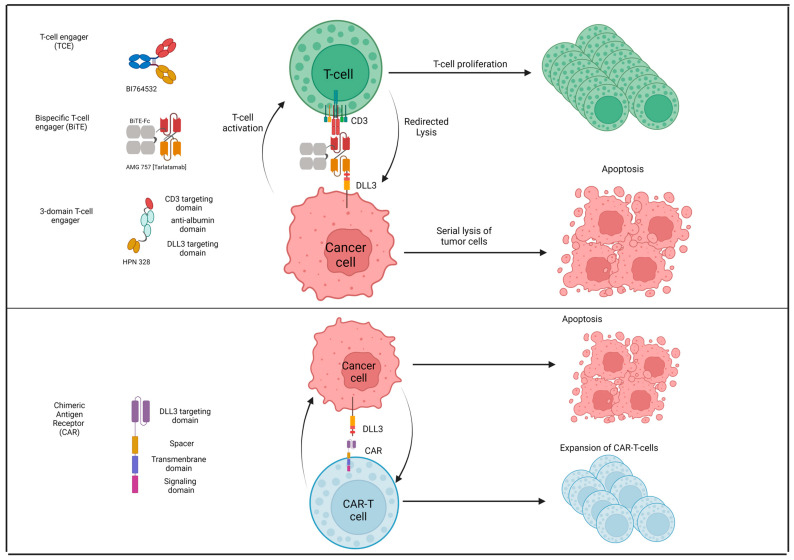
New anti-DLL3 agents in development in SCLC. Legend/abbreviations: DLL3 = delta-like ligand 3; BiTE = bispecific T-cell engager molecule; TCE = T-cell engager; CD3 = cluster of differentiation 3; CAR = chimeric antigen receptor; CAR-T = chimeric antigen receptor T-cell. Created with BioRender.com (accessed on 7 May 2024).

Chimeric antigen receptor (CAR) therapy is a new strategy in oncology; two phase I trials are ongoing with autologous CAR-T cells (NCT05680922) and CAR-NK cells (NCT05507593) to target DLL3 as reported in Table 5. Instead, Figure 4 depicts the new anti-DLL3 agents such as BiTE (Tarlatamab, BI-765432), TCE (HPN328) and CAR-cells.

In addition to the presented studies, we summarize in Table S17 the ongoing clinical trials with other strategies such as vaccines (including DC-vaccine [NCT04487756]), immune modulators (including bomedemstat, anti-LSD1 [NCT05191797], and radiation therapies (including TRIPLEX [CT05223647] and CHEST RT [NCT05796089] which are investigating chest radiotherapy in addition to CT + ICI, and RAPTOR [NCT04402788] investigating addition of radiotherapy on several tumor sites) [208].

## 6. Discussion

SCLC was first described a century ago in 1926 [209]. It is a separate entity from NSCLC and has been slowly recognized by biological and clinical studies, and by a different sensitivity to CT [210,211,212]. In the late 1970s, the therapeutic strategy (poly-CT) for advanced disease was defined until reaching the definitive standard of care based on PT/EP around the mid-1990s, and it remained so for decades [7,213,214,215,216,217]. Many approaches to overcome the results obtained from this two-drug combination had failed until the advent of ICIs: the addition of anti-PD-L1 (atezolizumab, durvalumab) but not anti-CTLA4 to standard CT was proven to prolong the overall survival by around two months with respect to CT alone, and more importantly for the first time in SCLC it allowed patients to reach a survival rate of 17.6% and 12% at 3 years and 5 years, respectively [9,10,18,20,21,22]. Subsequent randomized trials confirmed that adding an anti-PD-(L)-1 to CT can improve both PFS and OS [23,25,26,27,28]. Subgroup analyses investigating whether PD-L1 score or TMB could help to select patients who experienced the longer benefit with ICI were unfortunately negative [145,218,219] On the other hand, the low expression of MCH-I in SCLC (about 15%) helped us to recognize one of the main causes of ICI failure in SCLC in terms of immune system evasion [83]. Interestingly, in a preclinical experiment, bomedemstat (an LSD-1 inhibitor) increased MHC class I expression in mouse SCLC tumor cells and increased the response to ICI [220]. Therefore, it is necessary to develop strategies to increase the immunogenicity of SCLC, and research is going in that direction.

The treatment front for relapsed SCLC has been even more challenging, with few options available: topotecan, with higher responses in CT-sensitive SCLC patients; amrubicin approved only in Japan; and platinum–etoposide rechallenge clearly only possible for sensitive disease [48,49,50,51]. In June 2020, the FDA approved lurbinectedin for relapsed SCLC based on modest activity shown in a phase II basket trial. However, similar to the other chemotherapeutics, lurbinectedin performed best in patients with CT-sensitive disease [54]. The results from other chemotherapeutics (topoisomerase I inhibitors, alkylating agents, antimetabolites, new platinum compounds, taxanes) and formulations were even more disappointing, both in terms of efficacy and/or toxicity [121,122,123,124,125,126,127,128,129,130,131,132,133,134,135,136,137,138].

The addition of antiangiogenics or multi-tyrosine kinase to CT or ICI has given poor results in relapsed SCLC; the only interesting study with positive results was one in which anlotinib and benmelstobart were added to CT in the first-line setting; however, this trial is questionable given that it only included Chinese patients and used chemotherapy alone as a control arm [170]. Therefore, clinical trials using bispecific antibodies targeting VGFR and PD(L)-1 are ongoing.

There were great expectations of the PARPis; however, taking together the results from the various clinical trials, we can conclude that the activity of these drugs administered alone or in combination with different drugs (CT, ICI and mTKI) is poor in an unselected patient population [110,134,139,140,141,142,143,144]. Some studies reported that SLFN11 is a predictor of response to platinum and PARPis in SCLC [12,130,221]. Interestingly, a subgroup analysis from a randomized trial of temozolomide alone vs. temozolomide plus veliparib showed that patients whose tumors expressed SLNF11 experienced major benefit from the PARPi addition [130]. Consequently, the SWOG group designed a maintenance clinical trial testing the addition of a PARPi (talazoparib) to atezolizumab in patients with SCLC SLNF11+, confirming preliminary positive results [178].

Great hopes have always been placed on targeted therapies; however, disappointing results were obtained here also, both in unselected and in a biomarker-driven selected patient population [186]. In this context, nucleic acid therapy, such as RNA (si-RNA and mi-RNA), could represent a field of research that is still little explored. These small molecules could target genes if properly transported [222].

Another class of promising drugs are the ADCs, with fair results in relapsed SCLC. However, Rova-T in the randomized TAHOE phase III trial failed to demonstrate a survival benefit over topotecan in second-line in DLL3-high SCLC patients [191]. Nevertheless, the usefulness of DLL3 as a target is confirmed by the advent of a new generation of BiTE drugs (tarlatamab, BI 764532) which obtained promising results (ORR up to 40% in pretreated patients) [205,206] The strengths of these drugs can rely on the fact that they act irrespective of MHC class I expression. The ongoing randomized phase III DeLLphi-304 [NCT05740566] has been designed to compare tarlatamab versus investigator’s choice second-line chemotherapy. DLL3-CAR cells offer yet further promising therapeutics for this disease [223].

As we have seen, the history of SCLC is dotted with few successes and many failures. However, if the clinical results have been up to now poor or modest, we are witnessing a real revolution starting from translational research. In 2012, the double p53 and RB1 loss was discovered in mouse models [69,70]. In 2015, comprehensive genomic profiles of SCLC have been performed detecting, besides p53/RB1 loss, rare kinase gene mutations (potentially targets for new drugs) and inactivating mutations in NOTCH family genes in around 25% of SCLC [71]. In 2023, the largest molecular study identified wild-type RB1/TP53 tumors, SCLCs with STK11 mutation (also mutation in the PIK3CA and PTEN pathway were found), and SCLCs derived from transformation of NSCLC with different molecular characteristics [72]. Subsequently, between 2019 to 2024, phenotypic subtypes have been identified, with the two most representative having NE features (around 70%) (SCLC-A and SCLC-N) and the less common (around 30%) non-NE features (SCLC-P and SCLC-I) [110,112,224]. More importantly, the SCLC-I subtype has been identified as a potential target of ICI therapy, since it is associated with an inflamed phenotype (high expression of interferon-γ response genes, high 18-gene T-cell-inflamed gene expression profile score, and high expression of HLA and T-cell receptor genes) [111,114]. Recent findings have shown that patients who might benefit from the addition of ICI can be extracted from each SCLC subtype, since sensitivity to ICI is a balance between low TAM and high T-eff. This balance can be presented both in NE and non-NE SCLC subtypes [99].

Moreover, some studies have reported the temporal evolution of SCLC subtypes from NE to non-NE, suggesting that these are not distinct subtypes but different stages of progressive evolution [68,79]. The importance of these discoveries relies on the fact that we can modulate the SCLC phenotypes thanks to the administration of drugs such as LSD-1 or EZH2 inhibitor, as we reported earlier, with the aim to augment the sensitivity of patients with SCLC to ICI [205]. In fact, epigenetic modifications are the key contributors to the tumorigenesis of SCLC. A better understanding of epigenetic mechanisms may serve to better stratify patients, understand the mechanisms underlying chemoresistance and reduced sensitivity to immunotherapy, and better select potential targets for new therapies [87].

These findings can also help us to select patients for other treatments besides ICI: anti-DDL3 agents can be useful towards patients with non-inflamed NE SCLC subsets (SCLC-A and SCLC-N) having a high expression of DDL3, while patients with inflamed non-NE subsets (SCLC-P) may be sensitive to DNA-damaging drugs [119].

In conclusion, although the clinical results are not so encouraging, if we look at the bench research, it seems that we are moving slowly. We must reap the benefits of basic research and turn them into important breakthroughs in the treatment of SCLC patients.

## Figures and Tables

**Table 1:** Summary of clinical trials of CT plus ICI in treatment-naïve patients with ES-SCLC.

	IMPOWER-133	CASPIAN ^£^	ΚΕΥΝOΤΕ-604	ECOG-ACRIN EA516	ASTRUM-005	CAPSTONE-1	EXTENTORCH	RATIONALE-312
Phase	III	III	III	II	III	III	III	III
Place	Global	Global	Global	Global	China + 5 countries *	China	China	China
N (pts)	403	537	453	160	585	462	442	457
Drug (ICI)	Atezolizumab	Durvalumab	Pembrolizumab	Nivolumab	Serplulimab	Adebrelimab	Toripalimab	Tislelizumab
Class	Anti-PD-L1	Anti-PD-L1	anti-PD-1	Anti-PD-1	Anti-PD-1	Anti-PD-L1	Anti-PD-1	Anti-PD-1
Princ. Endp.	OS, PFS	OS	OS, PFS	PFS	OS	OS	PFS, OS	OS
CT/ICI Cycles (N) ^§^	4	4	4	4	4	4–6	4–6	4
CT arm Cycles (N) ^§,%^	4	6	4	4	4	4–6	4–6	4
Platins	Carboplatin	Carboplatin/Cisplatin	Carboplatin/Cisplatin	Carboplatin/Cisplatin	Carboplatin	Carboplatin	Carboplatin/Cisplatin	Carboplatin/Cisplatin
M phase	Atezolizuma/Placebo	Durvalumab/control	Pembrolizuma/Placebo	Nivolumab/observation	Serplulimab/Placebo	Adebrelimab/Placebo	Toripalimab/Placebo	Tislelizumab/Placebo
PS	0–1	0–1	0–1	0–1	0–1	0–1	0–1	0–1
PCI	Both arms	CT Arm	Both arms	Both arms	No	Both arms	No	No
mPFS								
CT/ICI	5.2 mos	5.1 mos	4.5 mos	5.5 mos	5.7 mos	5.9 mos	5.8 mos	4.7 mos
CT	4.3 mos	5.4 mos	4.3 mos	4.6 mos	4.3 mos	5.7 mos	5.6 mos	4.3 mos
HR [95% CI]	0.77 [0.62–0.96]	0.78 [0.65–0.94]	0.75 [0.61–0.91]	0.65 [0.46–0.91]	0.48 [0.38–0.9]	0.70 [0.57–0.86]	0.66 [0.53–0.82]	0.64 [0.52–0.78]
mOS								
CT/ICI	12.3 mos	13.0 mos	10.8 mos	11.3 mos	15.4 mos	15.3 mos	14.6 mos	15.5 mos
CT	10.3 mos	10.3 mos	9.7 mos	8.5 mos	10.9 mos	12.8 mos	13.3 mos	13.5 mos
HR [95% CI]	0.70 [0.54–0.91]	0.73 [0.59–0.91]	0.80 [0.64–0.98]	0.67 [0.46–0.98]	0.63 [0.49–0.82]	0.72 [0.58–0.90]	0.79 [0.64–0.98]	0.75 [0.61–0.92]
FDA	Yes	Yes	No	No	No	No	BD	No
EMA	Yes	Yes	No	No	Yes	No	No	No
Other					China	China	China	

Legend: ^£^ We summarized only two arms (CT plus durvalumab and CT plus placebo) and excluded the arm with CT plus durvalumab and tremelimumab. * ASTRUM-005 has been conducted in 6 countries: China, Georgia, Poland, Russia, Turkey, and Ukraine. ^§^ Number of cycles of CT/ICI or CT in the induction phase. ^%^ Control arm. Abbreviations: N (pts) = patient number; ICI(s) = immune checkpoint inhibitor(s); Princ. Endp. = principal endpoint(s); CT = chemotherapy; platins = platinum salts; M phase = Maintenance phase; PS = Eastern Cooperative Oncology Group (ECOG) Performance Status; PCI = prophylactic cranial irradiation; mPFS = median progression-free survival; HR = hazard ratio; CI = confidence interval; mOS = median overall survival; FDA = Food and Drug Agency; EMA = European Medicines Agency; PD-1 = programmed cell death protein 1; PD-L1 = programed death ligand 1; mos = months; BD = breakthrough designation.

**Table 2:** Features of Gay’s SCLC-subtypes.

Subtype	Phenotype	George Set (n = 81)	IMpower133 Set (n = 276)	TF Expression	Other Molecular Characteristics	Sensitiviness to Platinum and PARPi	Other Drug Sensitivity
SCLC-A	NE	36%	51%	High expression of ASCL1 and a low expression of NEUROD1	High expression of MYCL, SOX2, TTF-1, BCL2, CHGA, SYP, DLL3 and CEACAM5	Sensitive or refractory on the basis of SLFN11 expression (sensitive only with high expression of SLFN11)	Sensitive to BCL2 inhibitors and DLL3 agents
SCLC-N	NE	31%	23%	High expression of NEUROD1 and a low expression of ASCL1	Lacks of TTF1, high levels of INSM1,HES6, CHGA, SYP, DLL3 and SSTR2	Refractory to cisplatin and PARPi	Sensitive to AKI
SCLC-P	non-NE	16%	7%	High expression of POU2F3 and low expression of ASCL1 and NEUROD1	High expression of REST and c-MYC amplification. High expression of MICA (MCH I) *. Unexpressed DLL3	Sensitive but in this subgroup it is not linked with the expression of SLFN11, which is modest	Sensitive to PARPi, antimetabolites (anti-folates), and AKI
SCLC-I	non-NE	17%	18%	Low expression of ASCL1, NEUROD1 and POU2F3	High levels of mesenchymal markers (VIM and AXL). High expression of genes related to HLAs, interferon-γ activation, and immune checkpoints. High expression of BTK. Unexpressed DLL3	Refractory to cisplatin and PARPi	Sensitive to BTKi (e.g., ibrutinib), HDACi (e.g., mocetinistat) and ICIs

Legend: George set comprised LS-SCLC and Impower 133 set included ES-SLCL. * MICA: gene that encode major histocompatibility complex (MCH) class I polypeptide related sequence A. Abbreviation: TF = transcription factor; ASCL1 = achaete-scute homolog 1; NEURO = neurogenic differentiation factor; POU2F3 = POU class2 homeobox; CHGA = chromogranin A; SYP = synaptophysin; DLL3 = delta-like ligand 3; CECAM5 = carcinoembryonic antigen-related cell adhesion molecule 5; TTF1 = thyroid transcription factor-1; SSTR2 = somatostatin receptor 2; SOX2 = transcription factor sex determining region Y (SRY)-box 2; BCL2 = B-cell lymphoma 2; c-MYC = Avian Myelocytomatosis Viral Oncogene (v-myc) Homolog; MYCL = transcription factor member of the MYC family; INSM1 = insulinoma-associated protein 1; HES6 = transcription factor member of basic helix-loop-helix transcription repressor (bHLH); REST = RE1 silencing transcription factor; HLAs = human leucocyte antigens; VIM = vimentin; AXL = tyrosine protein kinase receptor UFO; PARPi = poly-ADP-ribose polymerase (PARP) inhibitors; BTK(i) = Bruton’s tyrosine kinase (inhibitors); HDACi = histone deacetylase inhibitors; AKI = Aurora kinase inhibitor; ICIs = immune checkpoint inhibitors.

**Table 3:** Features of Nabet’s SCLC subsets.

Subsets	Phenotype	TF Expression	Gay’s SCLC Subtype	Immune Composition	Benefit to IO (mOS with A-CP/ET vs. mOS with P-CP/ET)
MNF1	NE	Uniquely high NEUROD1 expression; high expression of ASLC1.	SCLC-N	T-eff-low/TAM-low	11.14 mos vs. 9.65 mos (HR 0.58; 95% CI: 0.36–0.95)
NMF2	NE	Highest expression of ASLC1.	SCLC-A	T-eff-low/TAM-high	10.84 mos vs. 10.02 mos (HR 0.79; 95% CI: 0.47–1.30)
NMF3	NE	High expression of ASLC1, YAP1 was elevated similar to NMF4, NOTCH3 mutations was enriched.	SCLC-I (NE) and SCLC-A	T-eff-high/TAM-low	16.37 mos vs. 8.63 mos (HR 0.45; 95% CI: 0.22–0.89)
NMF4	non-NE	POU2F3 uniquely expressed, low expression of ASCL1, YAP1 was elevated similar to NMF3, other non-NE drives such as REST and MYC were elevated compared to other NMF subsets.	SCLC-I (non-NE) and SCLC-P	T-eff-high/TAM-high	9.19 mos vs. 10.11 mos (HR 1.02; 95% CI: 0.55–1.91)

Abbreviation: TF = transcription factor; SCLC = small cell lung cancer; IO = immunotherapy; mOS = median overall survival; A-CP/ET = atezolizumab plus carboplatin plus etoposide; P-CP/ET = placebo plus carboplatin plus etoposide; ASCL1 = achaete-scute homologue 1; NEURO = neurogenic differentiation factor; POU2F3 = POU class2 homeobox; YAP1 = yes associated protein 1; NOTCH3 = Neurogenic locus notch homolog protein 3; REST = RE1-silencing transcription factor; T-eff = T-effector cells; TAM = tumor-associated macrophages; mos = months; vs. = versus; HR = hazard ratio; CI = confidence interval.

**Table 4:** Summary of clinical trials with ADCs in SCLC.

Reference	Ph	Setting	Drug	Target	Payload	N. SCLC	ORR	mPFS	mOS	Notes
TRINITY Morgensztern et al. [190]	II	≥3L	Rova-T	DLL3	PBD	339 [287 +] * [238 high] ^%^	12.4% 13.2% 14.3%	3.5 mos 3.8 mos 3.8 mos	5.6 mos 5.6 mos 5.7 mos	most common AEs any grade (G≥3 65%): fatigue 38%, photosensitivity reaction 36%, edema 31%. anorexia 30%
TAHOE Blackhall et al. [191]	III	2L	Rova-T vs. topotecan	DLL3	PBD	296 148	15% 21%	3 mos 4.3 mos	6.3 mos 8.3 mos	Rova-T was also more toxic than topotecan
MERU Johnson et al. [192]	III	M	Rova-T vs. placebo	DLL3	PBD	372 376	10% ^$^ 5% ^$^	4.7 mos 1.4 mos	8.5 mos 9.8 mos	AEs G>3 (59%): thrombocytopenia 9%, pleural effusion 4%, photosensitivity 4%
NCT01631552 IMMU-132-01 Gray et al. [196]	I	≥2L	Sacituzumab govitecan	TROP2	SN-38	50	14%	3.7 mos	7.5 mos	Benefit observed both in CT-sensitive and CT-refractory SCLC
IMMU-132-01 Bardia et al. [197]	I	≥2L	Sacituzumab govitecan	TROP2	SN-38	62	17.7%	3.7 mos	7.1 mos	Basket trial in all solid tumors
TROPiCS03 NCT03964727 Dowlati et al. [198]	II	2L	Sacituzumab govitecan	TROP2	SN-38	30	37% ^&^	NA	NA	Any AEs (G≥3 60%): diarrhea 67% (G3 7%), neutropenia 47% (G3 14%), constipation 43% (G3 0%), fatigue 43% (G3 3%), nausea 43% (G3 0%)
NCT03639194 Morgensztern et al. [199]	I	≥2L	ABBV-011	SEZ6	Calicheamicin	40 ^#^	25%	3.5 mos	NA	Any AEs (G≥3 65%): fatigue 48% (G3 10%), nausea 45% (G3 3%), anorexia 38% (G3 0%), platelet count decreased 38% (G3 10%), vomiting 35% (G3 3%)
IDeate-Lung01 NCT05280470 Johnson et al. [200]	II	≥2L	Ifinatamab Deruxtecan	B7-H3	Dxd	21	52.4%	5.8 mos	12.2 mos	Most common AEs (G≥3 36.4%): nausea and diarrhea

Legend: * DLL3 positive (+) patients: ≥25% of tumor cells positive for DLL3. ^%^ patients with high DLL3 (≥75% of tumor cells positive for DLL3). ^$^ Evaluated in DLL3 high patients. ^#^ Results of the dose expansion cohort. ^&^ median duration of response (DOR) = 6.3 months. Abbreviations: Ph = phase; Pt N = patient number; ORR = overall response rate; mPFS = median progression-free survival; mOS = median overall survival; 2L = second-line; 3L = third-line; M = maintenance; Rova-T = rovalpituzumab tesirine; DLL3 = delta-like ligand 3; SEZ6 = seizure-related homolog protein; TROP2 = trophoblast cell surface glycoprotein antigen 2; B7-H3 = B7 homolog 3 protein; PBD = pyrrolobenzodiazepine; SN-38 = analog of camptothecin; Dxd = deruxtecan; CT = chemotherapy; SCLC = small-cell-lung cancer; mos = months; AEs = adverse events; G = grade; NA = not available.

**Table 5:** Summary of ongoing clinical trials of new drugs directed towards DLL3 in SCLC patients.

Trial	Ph	Setting	Drug	Class	Pt N	ORR	mPFS	mOS	Notes
DeLLphi301	RII	≥3L	Tarlatamab 10 mg vs. 100 mg	BiTE	134 88	40% 32%	4.9 mos 3.9 mos	14.3 mos NR	CRS 51; ICANs 8% CRS 61%; ICANs 28%
NCT05361395	Ib	1L	Tarlatamab + anti-PD-L1 + CT	BiTE	340	-	-	-	Principal endpoint: safety Recruiting
DeLLphi-305 NCT06211036	III	M	Durvalumab ± Tarlatamab	BiTE	550	-	-	-	Principal endopoint: OS Recruiting
DeLLphi-304 NCT05740566	III	2L	Tarlatamab vs. lurbinectedin or topotecan or amrubicin	BiTE	700	-	-	-	Principal endpoint: OS Recruiting
NCT0442908	I	≥2L	BI764532	BiTE	107 57 SCLC	25% 39%	NR	NR	Any AEs, G ≥3 27% CRS (59%, G ≥3 (2%), LCD (20% G ≥3 (16%) dysgeusia (20% G ≥3 (0) asthenia (19 G ≥3 (1%) pyrexia (18% G ≥3 (0)
DAREON^TM^-5 NCT05882058	II	≥3L	BI764532	BiTE	120 *	-	-	-	Primary endpoint: ORR Recruiting
DAREON^TM^-8 NCT06077500	Ib	1L	BI764532 + anti-PD-L1 + CT	BiTE	60	-	-	-	Principal endpoint: DLTs Recruiting
NCT04471727	I/II	≥2L	HPN328	TCE	44 29 SCLC	-	-	-	Principal endpoint: safety and DLTs Recruiting
NCT05507593	I	≥2L	DLL3-CAR-NK cells	CAR	18	-	-	-	Principal endpoint: DLTs/MTD Recruiting
NCT05680922	I	≥2L	DLL3 autologous CAR-T-cells	CAR-T	41	-	-	-	Principal endpoint: RP2D Recruiting

Legend: * patients with high grade NET including SCLC; (+) = plus. Abbreviations: Ph = phase; Pt N = patient number; ORR = overall response rate; mPFS = median progression-free survival; mOS = median overall survival; M = maintenance; 1L = first-line; 2L = second-line; 3L = third-line; CT = chemotherapy; BiTE = bispecific T-cell engager molecule; TCE = T-cell engager; SCLC = small-cell lung cancer; mos = months; AEs = adverse events; CRS = cytokine release syndrome; ICANs = immune effector cell-associated neurotoxicity syndrome; LCD = lymphocyte count decreased; G = grade; CAR = chimeric antigen receptor cell; CAR-T = CAR = chimeric antigen receptor T-cell; DLTs = dose limiting toxicities; MTD = maximum tolerated dose; RP2D = recommended phase II dose; OS = overall survival.

## Data Availability

No new data were created or analyzed in this study. Data sharing is not applicable to this article.

## Supplementary Materials

The following supporting information can be downloaded at: https://www.mdpi.com/article/10.3390/genes15060701/s1. Table S1: Summary of Real-world clinical trials in first-line with CT plus ICI in patients with ES-SCLC. Table S2: Summary of studies with Topotecan in relapsed SCLC. Table S3: Summary of studies with platinum-etoposide rechallenge in patients with relapsed SCLC. Table S4: Results of clinical trials of lurbinectedin-based therapy in relapsed SCLC patients. Table S5: Ongoing clinical trials of lurbinectedin in ES-SCLC. Table S6: Summary of clinical trials with ICI in relapsed SCLC. Table S7: Survival according to subtypes in IMpower133. Table S8: Summary of clinical trials with chemotherapeutic agents in patients with relapsed SCLC. Table S9: Summary of studies with ICIs combinations in patients with relapsed SCLC. Table S10: Ongoing clinical trials with immunotherapy in patients with ES-SCLC. Table S11: Summary of clinical trials with apatinib (anti-VEGFR2) in patients with SCLC. Table S12: Summary of clinical trials multi-tyrosine kinase (mTKI) in patients with ES-SCLC. Table S13: Summary of ongoing clinical trials with antiangiogenics and combinations in patients with ES-SCLC. Table S14: Summary of clinical trials of PARP inhibitors in patients with ES-SCLC. Table S15: Clinical trials of targeted agents in patients with ES-SCLC. Table S16: Ongoing clinical trials with ADC in ES-SCLC. Table S17: Other ongoing trials in ES-SCLC [cancergov.trials].
