# Supplementary material for: The Evolving Scenario of ES-SCLC Management: From Biology to New Cancer Therapeutics
Source: Genes (Basel). 2024 May 27;15(6):701. doi: 10.3390/genes15060701 (PMC11203015; doi:10.3390/genes15060701)
Supplement: Supplementary file 1 [file genes-15-00701-s001.zip › genes-2993983-supplementary.pdf]

Supplementary Materials: The Evolving Scenario of ES-SCLC Management: From Biology to New Cancer Therapeutics

Pamela Trillo Aliaga[1*], Ester Del Signore[1], Valeria Fuorivia[1,2,3], Gianluca Spitaleri[1], Riccardo Asnaghi[1,2,3], Ilaria Attili[1], Carla Corvaja[1], Ambra Carnevale Schianca[1,2,3], Antonio Passaro[1], Filippo de Marinis[1]

Table S1: Summary of Real-world clinical trials in first-line with CT plus ICI in patients with ES-SCLC

	MAURIS	IMFIRST	CLINATEZO	ORIENTAL	LUMINANCE	CANTABRICO
**Trial ID**	NCT04028050	2019-002784-10*	NCT04920981	NCT04449861	NCT04774380	NCT04712903
**Recruitment status**	Completed<br>*(ESMO 2023 poster)*	Completed<br>*(ESMO 2023 poster)*	Completed<br>*(ASCO 2023 poster)*	Completed<br>*(ESMO 2023 poster)*	Active, Not recruiting<br>*(April 2024)*	Completed<br>*(ESMO 2023 poster)*
**Study phase**	IIIB	IIIB	Retrospective (EAP)	IIIB	IIIB	IIIB
**Treatment**	CBDCA+EP Atezolizumab (4/6 cycles)	CDDP/CBDCA+EP Atezolizumab (4/6 cycles)	CBDCA+EP Atezolizumab (4/6 cycles)	CDDP/CBDCA+EP Durvalumab (4/6 cycles)	CDDP/CBDCA+EP Durvalumab (4/6 cycles)	CDDP/CBDCA+EP Durvalumab (4/6 cycles)
**n (pts)**	155	155	518	166	152	101
**ECOG PS**	0-2	0-2	0-2	0-2	0-2	0-2
**PCI allowed**	Yes	Yes	Yes	Yes	Yes	No
**Chest RT allowed**	Yes	Yes	Yes	No	No	No
**1°endpoint**	Incidence G≥3 AE ir-AE	Incidence G≥3 AE ir-AE	Incidence G≥3AE ir AE	Incidence G≥3 AE ir AE	Incidence G≥3 AE ir AE	Incidence G≥3 AE ir-AE

Legend: * EudraCT Number. **Abbreviations:** N (pts) = patient number; ID= identification; PS = Eastern Cooperative Oncology Group (ECOG) Performance Status; PCI = prophylactic cranial irradiation; RT = radiotherapy; CBDA = carboplatin; EP = etoposide; CDDP = cisplatin; G = grade; AEs = adverse events; ir-AEs = immune-related adverse events; ESMO = European Society of Medical Oncology; ASCO = American Society of Clinical Oncology.

Table S2: Summary of studies with Topotecan in relapsed SCLC

TOPOTECAN<br>History Trials	Topotecan IV<br>vs CAV<br>von Pawel et al.<br>(JCO 1999)	Topotecan OS<br>Vs Topotecan IV<br>von Pawel et al.<br>(JCO 2001)	Topotecan OS<br>vs BSC<br>O'Brien et al.<br>(JCO 2006)	Amrubicin<br>vs Topotecan IV<br>von Pawel et al.<br>(JCO 2014)
Study Phase	III	II	III	III
Line of treatment	2	2	2	2
n (pts)	211 (S)	106 (S)	141 (S/R)	637 (S/R)
ORR (%)	24% (T)<br>18% (CAV)<br>p = .285	23% (OS)<br>95% CI 11.6-34.5<br>15% (IV)<br>95% CI 5.3-24.3	NR (BSC)<br>7% (T) → >R	31% (A)<br>17% (T)<br>OR= 2.223; p < .001
mPFS (mos)	3.2 mos (T)<br>3.0 mos (CAV)<br>p = .552	3.7 mos (OS)<br>3.2 mos (IV)	NR (BSC)<br>4 mos (T)	4.1 mos (A) >S<br>3.5 mos (T) >S<br>HR 0.802; p = .018
mOS (mos)	6.2 mos (T)<br>6.2 mos (CAV)<br>p = .795	8 mos (OS)<br>6.2 mos (IV)<br>HR 0.84 (95% CI, 0.53-1.32)	3.5 mos (BSC)<br>4.0 mos (T)	7.5 mos (A) >S<br>7.8 mos (T) >S<br>HR 0.880; p = .170
Grade ≥3 toxicity (%)				
Anemia	42% (T), 20% (CAV)	31% (OS), 30% (IV)	NR (BSC), 25% (T)	16% (A), 31% (T)
Thrombocytopenia	58% (T), 15% (CAV)	53% (OS), 49% (IV)	NR (BSC), 38% (T)	21% (A), 54% (T)
Neutropenia	89% (T), 87% (CAV)	57% (OS), 94% (IV)	NR (BSC), 61% (T)	41% (A), 54% (T)
FDA approval	Topotecan IV<br>1998		Topotecan OS<br>2007	

Abbreviations: *n (pts)* =patient number; R= resistant; S= sensitive. ORR = overall response rate; mPFS = median Progression Free Survival; mOS = Median Overall Survival; mos= months; T = Topotecan; A = amrubicin; CAV = vincristine, adriamycin, cyclophosphamide; BSC = Best Supportive Care; OS = Oral Administration; IV = Intravenous; HR= Hazard Ratio; OR= Odds Ratio; NR=not reported.

Table S3: Summary of studies with platinum-etoposide rechallenge in patients with relapsed SCLC

Reference	Phase	Schedule	Pt N	ORR	mDoR	MS
**Giaccone 1987**	II	VAC<br>VAC/CE<br>CDE	13	50%	22 weeks	23.5 months
**Genestreti 2015**	Retr.	CE	112	45%	5.5 months*	7.9 months
**Naito 2018**	Retr.	CE	67	52.2%	5.1 months*	10.8 months
**Baize 2020**	III	CE	86	49%	5.4 months	7.5 months

Legend * we stated mPFS (median progression-free survival) on behalf of mDoR because it was not reported. **Abbreviations:** Pt N=patient number; ORR = overall response rate; mDoR = Median duration of response; MS = Median survival; Retr. = retrospective; VAC = vincristine, adriamycin, cyclophosphamide; CE = cisplatin/etoposide; CDE = cyclophosphamide, doxorubicin and etoposide.

Table S4: Results of clinical trials of lurbinectedin-based therapy in relapsed SCLC patients

Reference		Phase	Schedule	Pt N	ORR		mPFS		AEs<br>G> 3 rate
**Trigo el al. 2020**		II	Lurbinectedin<br>3.2 mg/sm q21 days	105	35.2%		3.5 mos		NR
R<br>D <90	S<br>D ≥90				12%	45%	2.6 mos	4.6 mos	
**Ponce-Aix et al 2021**		I-II	Lurbinectedin 2 mg/sm q21 plus irinotecan 75mg/ms 1,8q21 days (+GCSF)	21	62%		6.2 mos		28.6 %<br>G4 neutropenia<br>9.5%<br>Febrile neutropenia
R<br>D <90	S<br>D ≥90				50%	69%	3.7 mos	8.5 mos	
**Aix et al. 2023***		III	lurbinectedin 2 mg/m2 plus doxorubicin 40 mg/m2 q 21 days	307	32%		4 mos		48%
R<br>D <180	S<br>D ≥180				NR	NR	4.5 mos	8.2 mos	
**Calles Blanco et al. 2023**		II	Lurbinectedin<br>3.2 mg/sm plus pembrolizumab 200 mg q21 days	28	46.4%		5.3 mos		82.1%
R<br>D <90	S<br>D ≥90				35%	54%	3 mos	10 mos	

Abbreviations: mg = milligrams; sm = square meter; q = every; Pt N = patient number; ORR = overall response rate; mPFS = median progression-free survival; AEs= adverse events; G = grade; R= resistant; S=sensitive; D=days; mos= months; GSCF= Granulocyte colony-stimulating factor; NR= not reported.

Table S5: Ongoing clinical trials of lurbinectedin in ES-SCLC

N. Identifier	Phase	Drug(s)	Disease	Pt N	Principal endpoint(s)	Notes
**IMforte NCT05091567**	III	Lurbinectedin + atezolizumab vs atezolizumab	M SCLC	450	PFS/OS	Key eligibility criteria: No BM<br>SFs are: ECOG PS 0 vs 1<br>Liver metastases y vs n<br>LDH ULN > vs <<br>PCI y vs n
**NCT02611024**	I/II	Lurbinectedin + irinotecan	Relapsed Solid tumors	320	RP2D/ORR	Aix SP 2019: phase I ORR (3/11) 27%<br>The RD is LUR 2.0 mg/m2 on D1 + CPT11 75 mg/m2 D1,8 q21
**LAGOON NCT05153239**	III	Lurbinectedin +irinotecan vs Lurbinectedin vs irinotecan or topotecan	2L SCLC	705	OS	Key eligibility criteria:<br>CTFI > 30 days<br>SFs are: CTFI < or > 90 days<br>Prior ICI y vs n<br>LDH ULN > vs <<br>BM y vs n<br>Topotecan vs irinotecan
**NCT04607954**	II	Durvalumab + topotecan or lurbinectedin	Relapsed SCLC (after CT/IO)	70	ORR	Group 1 SCLC sens. D + T<br>Group 2A SCLC sens D+ L<br>Group 2B SCLC refr. D+L
**LURBIMUNE NCT05572476**	II	Durvalumab + lurbinectedin	Relapsed SCLC	82	ORR	Sensitive SCLC; non comparative ARM B<br>Rechallenge CT
**NCT04253145**	I/II	Atezolizumab+ lurbinectedin	Relapsed SCLC	184	ORR	After CT or CT/IO
**NCT05572476**	Ib	Lurbinectedin + RT	Relapsed SCLC	22	DLT/ORR	After CT or CT/IO
**NCT05578326**	II	Lurbinectedin + trilaciclib	Relapsed SCLC	30	ORR	Trilaciclib is a myelopreservation drug
**NCT04802174**	I/II	Lurbinectedin + Berzosertib	Relapsed tumor	75	DLT/ORR	High grade NE tumors<br>Berzosertib ATR inh<br>At least one line CT

Abbreviations: Pt N = patient number; RT = radiotherapy; M = maintenance; SCLC = small cell lung cancer; 2L = second line; CT = chemotherapy; IO = immunotherapy; NE = neuroendocrine tumor; BM = brain metastases; PFS = progression-free survival; OS = overall survival; SFs = stratification factors; ECOG PS = Eastern Cooperative Oncology Group performance status; ULN = upper limit normal; PCI = Prophylactic cranial irradiation; ORR = overall response rate; RD = recommended dose; sens. = sensitivity; refr = refractory; ATR = Ataxia telangiectasia and Rad3; inh. = inhibitor

Table S6: Summary of clinical trials with ICI in relapsed SCLC

	KEYNOTE-028 Ott et al., JCO 2017	KEYNOTE-158 Chung et al., JCO 2018	IFCT-1603 Pujol et al., JTO 2019	CHECKMATE-032 Ready et al., JTO 2019	CHECKMATE-331 Spigel et al., Ann Onc 2021
**Drug**	Pembrolizumab (only Pdl1+)	Pembrolizumab (any Pdl1)	Atezolizumab vs CT	Nivolumab +/- Ipilimumab	Nivolumab Vs Topot. or Amrub.
**Line**	2/3	2/3	2	2/3	2
**Phase Study**	IB (basket)	II (basket)	II	I/II	III
**n (pts)**	24	107 (42 Pdl1+/50 Pdl1-)	73 (49 A, 24 CT)	147 (N) 96 (N+I)	781 (284 N, 285 CT)
**1°endpoint**	ORR	ORR	ORR	ORR	OS
**ORR (%)**	33.3% [95% CI, 16-55]	18.7% (O) [95% CI, 11.8–27.4] 35.7% (Pdl1+) 6% (Pdl1-)	2.3% (A) [95% CI: 0.0–6.8] 10% (CT) 95% CI: 0.0–23.1	11.6% (N) 21.9% (N+I) OR: 2.12 (95% CI 1.06–4.26)	13.7% (N) 16.5% (CT) OR 0.80 (95% CI, 0.50-1.27)
**mPFS (mos)**	1.9 mos [95% CI, 1.7-5.9]	2.0 mos (O) [95% CI, 1.9-2.1] (2.1 Pdl1+, 1.9 pdl1-)	1.4 mos (A) [95% CI, 1.2-1.5] 4.3 mos (CT) [95% CI, 1.5-5.9]	1.4 mos (N) [95% CI, 1.3–1.4] 1.5 mos (N+I) [95% CI, 1.4–2.2]	1.4 mos (N) 3.8 mos (CT) HR 1.41 (95% CI, 1.18-1.69)
**mOS (mos)**	9.7 mos [95% CI, 4.1-not reached]	9.1 mos [95% CI, 5.7-14.6] (14.6 Pdl1+, 7.7 Pdl1-)	9.5 mos (A) 8.7 mos (CT) HR 0.84 (95% CI, 0.45-1.58)	5.7 mos (N) [95% CI, 3.8-7.6] 4.7 mos (N+I) [95% CI, 3.1 to 8.3]	7.5 mos (N) 8.4 mos (CT) HR 0.86 (95%CI, 0.72-1.04, p 0.11)
**DoR (mos)**	19.4 mos >3.6 to >20.0	NR	NR	15.8 mos (N) [95% CI, 7.4– not reached] 10.0 mos (N+I) [95% CI, 6.7–not reached]	8.3 mos (N) [95% CI, 7.0-12.6] 4.5 mos (CT) [95% CI, 4.1-5.8]

Abbreviations: ICI= immune checkpoints inhibitors; n (pts) =patient number; ORR = overall response rate; mPFS = median Progression Free Survival; mOS = Median Overall Survival; DoR = Duration of Response; CT = Chemotherapy; O=0verall population; A = Atezolizumab; N = Nivolumab ; I = Ipilimumab; CI= confidence interval; OR= Odds ratio; HR= Hazard ratio; mos=months. NR= not reported.

Table S7: Survival according to subtypes in IMpower133*

Subtypes	phenotype	EP+ A arm		EP+ placebo arm		HR (95% CI)
		n.	mOS (months)	n.	mOS (months)	
**SCLC-A**	NE	77	10.9	63	10.6	0.807 (0.547-1.189)
**SCLC-N**	NE	25	10.6	36	9.4	0.631 (0.353-1.129)
**SCLC-P**	non-NE	9	9.6	12	6.0	0.595(0.223-1.589)
**SCLC-I**	non-NE	21	18.2	28	10.4	0.572 (0.284-1.15)
**All**	/	132	11.6	139	10.1	/

Legend: * Analysis reported in the publication of Gay's at al. **Abbreviations:** N (pts) =patient number; mOS = Median Overall Survival; HR = Hazard Ratio; CI= confidence interval; NE = Neuroendocrine; non-NE= non neuroendocrine. EP= etoposide/carboplatin chemotherapy; A = atezolizumab; SCLC= small cell lung cancer.

Table S8: Summary of clinical trials with chemotherapeutic agents in patients with relapsed SCLC

Reference	Setting	Phase	Schedule	Pt N	ORR	mPFS	MS	Notes
**Topoisomerase I inhibitors**								
**Goto 2016**	2L	III	CPT11 + CE<br>vs<br>topotecan	180	84%<br>vs<br>27%	5.7 mos<br>vs<br>3.6 mos	18.2 mos<br>vs<br>12.5 mos	Severe toxicity
**Kondo 2018**	≥2L	II	CPT11	30	41.3%	4.1 mos	10.4 mos	-
**Alamgeer 2018**	2L	II	CPT11/Hyaluronic + CBDCA	13	22%	7 mos	3.5 mos	-
**Arnold 2020**	2L	II	CPT11+ carfilzomib	62	21.6%	3.6	6.9 mos	-
**Zhao 2020**	2L	II	CPT11+ apatinib	17	40%	-	-	-
**Edelman 2020**	2L	II/III	Dinutuximab + CPT11<br>vs<br>CPT11 or topotecan	471	17.1%<br>vs<br>18.9%	3.5 mos<br>vs<br>3 mos	6.9 mos<br>vs<br>7 mos	Negative trial
**Spigel 2021 [RESILIENT]**	2L	II/III	Liposomal CPT11	30	44%	4 mos	8 mos	Phase III controlled vs topotecan negative
**Kang 2021**	2L	R II	Belotecan<br>Vs<br>topotecan	164	33%<br>Vs<br>21%	4.8 mos<br>Vs<br>3.8 mos	13.2 mos<br>Vs<br>8.2 mos	Belotecan was favorable for Pts < 65 years
**Alkylating agents**								
**Tanaka 2018**	2L	II	Ifosfamide	12	0%	0.9 mos	4.8 mos	Negative trial
**Pietanza 2018**	2-3L	R II	TMZ + veliparib<br>vs<br>TMZ	104	39%<br><br>14%	3.8 mos<br><br>2 mos	8.2 mos<br><br>7 mos	Plus PARP inhibitor
**Goldman 2022**	≥2L	II	Low dose TMZ + Talazoparib	28	39.3%	4.5 mos	11.9 mos	Plus PARP inhibitor
**Other chemotherapeutic agents**								
**Zheng 2016**	≥2L	II	Amrubicin	95	39%	NR	9.2 mos	-
**Yoshioka 2018**	≥2L	II	Weekly amrubicin	21	19%	4 mos	10 mos	Lack of efficacy
**Moharana 2016**	2L	IV	Paclitaxel	NR	9.5%	1.5 mos	12.5 mos	Designed for CT refractory
**Scagliotti 2016**	2L	R II	TAS-102<br>vs<br>Topotecan or amrubicin	18	0	1.4 mos<br>vs<br>2.7	NR	Lack of efficacy
**Gelsomino 2020 [NABSTER]**	2L	II	Nabpaclitaxel	68<br>S 47<br>R 25	 14%<br>8%	 1.9 mos<br>1,8 mos	 6.6 mos<br>3.6 mos	 Negative trial
**Cheng 2021**	2L	IV	Lobaplatin-based CT	431	33%	4 mos	9.2 mos	Real world data in China-

Abbreviations: Pt N = patient number; ORR = overall response rate; mPFS = median progression-free survival; MS = median survival; CPT11 = irinotecan; NA not applicable; mos = months; TMZ= temozolomide; 2L = second-line setting; CT = chemotherapy; S = sensitive; R refractory.

Table S9: Summary of studies with ICIs combinations in patients with relapsed SCLC

Reference	Setting	Drug(s)	Phase	Pt N	ORR	mPFS	mOS	Notes
PD-1 inhibitors								
**Kim 2019**	2L	Pembrolizumab + paclitaxel	II	26	23%	5 mos	9.1 mos	-
**Fischer 2019 [BIOLUMA]**	2L	Nivolumab + Ipilimumab	II	18	38.8%	-	-	Trial emendated to select highTMB
**Lycan 2021**	2L	Nivolumab + gemcitabine	II	14	8%	1.8 mos	3.2 mos	Closed due to futility
**Akamatsu 2021**	≥2L	Pembrolizumab + amrubicin	II	25	52%	4 mos	-	-
**Ozuma 2023**	2L	Nivolumab + LF eribulin	Ib/II	34	24%	4 mos	NR	mFUP 10.6 mos
**Fan 2020 [PASSION]**	2L	Camrelizumab + Apatinib	II	59	34%	3.6 mos	8.4 mos	-
**Zhang 2021**	2L	Penpulimab + Anlotinib	II	20	50%	4.7 mos	-	-
PD-L1 inhibitors								
**Thomas 2019**	2L	Durvalumab + olaparib	II	20	10.5%	1,8 mos	4,1 mos	
**Pakkalas 2020**	≥2L	Durvalumab + Tremelimumab +/- SRT	RII	18	0% vs 28.6%	2.1 mos vs 3.3 mos	2.8 mos vs 5.7 mos	
Anti-TIGIT								
**Rudin 2023** SKYSCRAPER-02	1L	Tiragolumab +/- CE+ Atezolizumab	III	490	73.5% vs 66.7%	5.4mos vs 5.6 mos	13.1 mos vs 13.1 mos	G3/4 AEs 64.0% vs 63.8%
Anti-LAG3								
**Uboha 2019**	≥2L	Spartalizumab (antiPDL1) + LAG525 (anti-LAG3)	II	76 (15*)	NR	NR	NR	Solid tumors

Abbreviations: Pt N = patient number; ORR = overall response rate; mPFS = median progression-free survival; mOS = median overall survival; PD-1 = Programmed Cell Death Protein 1; PD-L1 = PD-1 ligand 1; 2L = second-line; R II= randomized phase II trial; LP = liposomal; CE = cisplatin/carboplatin etoposide; SRT = stereotactic radiotherapy; mos = months; TMB = tumor mutational burden; mFUP = median follow-up; LDH = Lactate dehydrogenase; UNL = upper normal limit; mets = metastases; G=grade; AEs=adverse events: NR= not reported.

Table S10: Ongoing clinical trials with immunotherapy in patients with ES-SCLC

Identifier	Phase	Pt N	Drugs	Setting	Principal Aim	Note
**ASTRIDE** **NCT05468489**	III	200	CE+ atezolizumab vs CE + Serplulimab (anti-PD1)	1L	OS	Recruiting USA
**NCT04346914**	Ib	20	Socazolimab [ZKAB001] (anti-PDL1) + CT	1L	DLT	Recruiting China

**NCT04702880**	R II	120	BMS-986012 (antiFucosyl-GM1)$^{\$}$ + CT+ Nivolumab	1L	AEs	Not Recruiting<br>USA, Canada, Europe, Japan, Australia
**NCT05309629**	II	40	CE+ QL1706 (bispecific antibody anti-PDL1/anti-CTL4)	1L	Safety	Not Recruiting
**NCT05844150**	II/III	445	PM8002 (bispecific antibody anti-PDL1/anti-VEGF-A) + CT<br>vs<br>atezolizumab + CT	1L	ORR/OS	Recruiting<br>China
**KEYMOTE-B99**<br>**NCT04924101**	II	120	pembrolizumab + CP/EP + MK-4830 (anti-ILT4 antibody) + boserolimab (anti-CD27 antibody) or lenvatinib	1L	ORR/6-mos PFS	Active, not recruiting
**MOZART**<br>**NCT05903092**	II	38	Monalizumab<br>(anti HLA-E)<br>+ CT + Durvalumab	1L	1-yr PFS	Recruiting<br>USA
**TAXIO**<br>**NCT05856695**	II	67	Carbo + taxolo + durvalumab	1L	OS	Etoposide CT free<br>Recruiting<br>France
**EDUR-BRA**<br>**NCT06008353**	IV	60	Durvalumab + CT	1L	Observational	RWD<br>Recruiting<br>Brazil
**DRIVE**<br>**NCT05761977**	IV	50	Durvalumab + CT	1L	Observational	RWD<br>Recruiting<br>Greece
**ARSENAL**<br>**NCT05683977**	IV	250	Durvalumab + CT	1L	TTD	RWD<br>Recruiting<br>France
**NCT05191797**	I/II	34	Bomedemstat (anti LSD1) +<br>Atezolizumab	M	DLT	Recruiting<br>USA
**NCT05901584**	II	30	Cadonilimab [AK104] (bispecific antibody anti-PD1/anti-CTL4)<br>+/- 2L CT*	≥2L	PFS	Recruiting<br>China
**NCT05505825**	I/II	42	AK104 (antiPD1/CTLA4)<br>+<br>Chiauranib (mTKI)	2L	ORR	Recruiting
**NCT04085185**	Ia/Ib	268	IBI110 (anti-LAG3) +/- sintilimab	2L	safety	Solid tumors<br>(include SCLC)

						Recruiting
**NCT06016270**	I/II	130	nelmastobart [hSTC810] (anti-BTN1A1)$ + PTX	2L	ORR/PFS	Recruiting USA/Korea
**NCT05199272**	I/II	141	23ME-00610 (anti-CD200R1)$	≥2L	DLT	Solid tumors (include SCLC), Recruiting USA Canada

Legend: *Platinum based chemotherapy; $Humanized monoclonal antibody; GM1: ganglioside; **Abbreviation:** CE=carboplatin/etoposide; CT=chemotherapy; 1L= first line; 2L= second line; DLT=dose limiting toxity; ORR=overall response rate; OS= overall survival; PFS= progression free survival; VEGF-A= vascular endothelial growth factor A; HLA-E =human leucocyte antigen-E; LSD1 = Lysine-specific demethylase 1; SCLC=small cell lung cancer; RWD= real world data; PTX= paclitaxel.

Table S11: Summary of clinical trials with apatinib (anti-VEGFR2) in patients with SCLC

Reference	Phase	Drug	Pt N	ORR	mPFS	MS
Xu 2019	II	Apatinib	40	17.5%	3 mos	5.8 mos
Liu 2019	II	Apatinib	22	13.6%	5.4 mos	10 mos
Hua 2019	IV	Apatinib	52	19.4%	6.18 mos	-
**Phase II trials of combo**						
Zhao 2020	II	Apatinib +CPT11	17	40%	-	-
Fan 2020 PASSION	II	Apatinib+ Camrelizumab (anti-PD1)	59	34%	3.6 mos	8.4 mos
He 2021	II	Apatinib+ Vepesid	53	21%	3 mos	5 mos
Ma 2021	II	Apatinib + CPT11 or TXT	31	25%	7.4 mos	12.5 mos

Abbreviations: Pt N: patients number; mPFS: median progression-free survival; ORR: Overall Response Rate; MOS: Months;CPT11: Irinotecan; TXT: Docetaxel

Table S12: Summary of clinical trials multi-tyrosine kinase (mTKI) in patients with ES-SCLC

Reference	Phase	setting	Drug	Target(s)	Pt N	ORR	mPFS	M=S
Cheng 2021 [ALTER 1202]	R II	≥2L	Anlotinib vs placebo	VEGFR PDGFR FGFR c-kit	120	4.9% vs 2.6%	4.1 mos vs 0.7 mos	7.3 mos vs 4.9 mos
Wang 2021 [SALTER]	II	≥2L	Anlotinib + S1		52	43.8%	4.8 mos	6.3 mos
Zhang 2021	II	≥2L	Anlotinib + penpulimab (anti-PD1)		20	50%	4.7 mos	-
Zhang 2023	II	≥2L	Anlotinib+ TXT or CPT11		36	58%	4.4 mos	7.9 mos
Cheng 2023	III	1L	Anlotinib+ Benmelstobart (anti-PD-L1) + CP/EP vs CP/EP + placebo		493	81% vs 66%	6.9 mos vs 4.2 mos	19.32 mos vs 11.89 mos
Koinis 2017	II	≥2L	Pazopanib	VEGFR PDGFR cKIT	58	13.8%	2.5 mos	6 mos
Sun 2018 KCSG-LU12-07	R Ph II	≥2L	Pazopanib		95	NA vs NA	3.7 mos vs 1.8	10.6 mos vs 12.9 mos
Shi 2021	II	≥2L	Chiauranib	Aurora B VEGFR c-KIT CSF1-R	28	17.9%	3.6 mos	8.4 mos

Abbreviations: Pt N: patients number; mPFS: median progression-free survival; mOS: median overall survival; CP: carboplatin; EP:etoposide; NR: not reported; S1: oral dihydropyrimidine dehydrogenase (DPD) inhibitory fluoropyrimidine; VEGFR: Vascular Endothelial Growth Factor Receptor; FGFR: Fibroblast Growth Factor Receptor; PDGFR: Platelet Derived Growth Factor Receptor; c-KIT: Proto-oncogene receptor Tyrosine Kinase; NA: Not Available; mos: months; TXT: Docetaxel; CPT11: Irinotecan; CSF1-R: colony stimulating factor receptor

Table S13: Summary of ongoing clinical trials with antiangiogenics and combinations in patients with ES-SCLC

Identifier	Phase	Setting	Pt N	Drugs	Principal Aim	Note
**BELIEVE NCT05588388**	II	1L	39	CT+Atezolizumab + Bevacizumab	6-moPFS rate	Pts with liver mets Not yet recruiting USA
**CeLEBrATE**	II	1L	53	CT+ Atezolizumab + Bevacizumab	1year-OS rate	Active, recruiting
**NCT05668767**	II	1L	20	CT+ Durvalumab + Surufatinib$	PFS	Recruiting China
**NCT05882630**	I/II	1L	39	CT+ Serplulimab (antiPD1) +Surufatinib$	PFS	Recruiting China
**NCT04660097**	II	1L	120	CT+ Durvalumab + Anlotinib	OS	Recruiting China
**PEERS NCT05384015**	II	1L	85	CT+ Pembrolizumab + Lenvatinib	AEs/PFS	Recruiting Spain
**NCT04683198**	II	1L	68	CT+ Camrelizumab (antiPD1) +Apatinib	PFS	Recruiting (last updated 24 Dec 2020) China

**NCT05116007**	Ib	1L	35	AK112 (BiAb anti-PD1/antiVEGF)+ CT	AEs, ORR	Active, not recruiting
**NCT05896059**	II	M	21	Tislelizumab (anti-PD1) +Anlotinib	1-yr PFS	Recruiting China
**NCT05509699**	II	M	60	ICI*+Surufatinib$	PFS	Recruiting China
**NCT05505825**	I/II	2L	42	AK104 (BiAb antiPDL1/CTLA4) + Chiauranib	ORR	Recruiting China/Australia
**NCT05296603**	II	2L	83	IBI-322 (BiAb antiPDL1/CD47) + Lenvatinib	ORR	Recruiting China

Legend: *anti-PD (L)-1 inhibitor; $ small molecule, multi-tyrosine kinase inhibitor against VEGFR/FGF1R/CSF1-R. **Abbreviation**: CT=platinum plus etoposide chemotherapy. BiAb= bispecific antibody; PFS= progression free survival, OS= overall survival; AEs= adverse events; ORR= overall response rate.

Table S14: Summary of clinical trials of PARP inhibitors in patients with ES-SCLC

Reference	Setting	Phase	Schedule	Pt N	ORR	mPFS	MS
**Pietanza 2018**	2L	R II	TMZ + Veliparib vs TMZ+placebo	104	39% vs 14%	3.8 mos vs 2.0 mos	8.2 mos vs 7.0 mos
**Owonikoko 2019**	1L	R II	C/E+Veliparib Vs C/E+placebo	128	71.9% vs 65.6	6.2 mos vs 5.5 mos	10.3 mos vs 8.09 mos
**Thomas 2019**	2L	II	Olaparib + Durvalumab	20	10.5%	1,8 mos	4,1 mos
**Kim 2020**	2L	II	Olaparib + Cediranib (VEGFR-I)	25	28%	4.1 mos	5.5 mos
**Park 2021 SUKSES-B**	2L	II	Olaparib	15	6.7%	1.25 mos	8.56 mos
**Park 2021 SUKSES-N2**	2L	II	Olaparib+ Ceralasertib (ATR-I)	26	3.8%	2.75 mos	7.18 mos
**Fan 2021**	2L	Ib	Fluzoparib + Adebrelimab (PD-L1)	23	4.3%	1.4 mos	6.7 mos
**Huang 2023 TRIDENT**	M	II	Olaparib + durvalumab	60	NA	5.8 mos	NR
**Karim 2023 SWOG S1929**	M	R II	Atezolizumab + talazoparib vs Atezolizumab	106	16% vs 12%	4.2 mos vs 2.8 mos	9.4 mos vs 8.5 mos

Ongoing clinical trials						
ID	Setting	Phase	Therapy	N. Pt	Principal Endpoint	Notes/status
THOR NCT05623319	1L	II	Olaparib+ Pembrolizumab	60	mPFS	Recruiting Italy
NCT06217757	1L	I/II	Olaparib + sugemalimab + CT + low dose RT	45	RD	SLFN-11 Positive ES-SCLC Not yet recruiting China
PRIO trial NCT04728230	1L	I/II	Olaparib + durvalimab + CT	63	DLT	Recruiting USA
NCT03830918	M	Ib/II	Niraparib + atezolizumab + TMZ	74	RD/PFS	Recruiting USA
RAISE NCT05718323	M	II	Niraparib + AntiPD-L1	44	PFS	*Pts with SLFN11+* Not yet Recruiting Europe
NCT04782089	M	Ib	Camrelizumab Fluzoparib	20	PFS	Not yer recruiting Last update 4 Mar 2021
NCT04170946	M	Ib	Talozoparib + low dose RT	24	MTD	Recruiting Canada
NCT05975944	2L	I/II	Olaparib + selinexor (anti-JAK)	49	MTD/ORR	Recruiting China
TRIO-US L-07 NCT03672773	2L	II	Talazoparib + low dose TMZ	28	ORR	Recruiting USA
NCT05728619	≥2L	I/II	HTMC0435 + TMZ	64	DLT	Recruiting China

Abbreviations: Pt N = patient number; ORR = overall response rate; mPFS = median progression-free survival; MS = median survival; 2L = second-line setting; M= maintenance; TMZ = temozolomide; VEGFR-I = Vascular Endothelial Growth Factor-inhibitor; ATR= Ataxia-telangiectasia and Rad3 related; Atezo = atezolizumab; mos = months; P=cisplatin; E=etoposide.

Table S15: Clinical trials of targeted agents in patients with ES-SCLC

Reference	Ph	Setting	Drug	Target	Pt N	ORR	mPFS	mOS	Notes
Chiappori 2016	R II	2L	Linsitinib vs topotecan	IGF1R	29<br>15	0%<br>13%	1.2 mos<br>3 mos	3.4 mos<br>5.3 mos	*Lack of efficacy*
Awad 2017	II	2L	BI2536	PLK1/BRD4	23	0	1.4 mos	7.8 mos	Trial was designed for CT-sensitive SCLC. *Lack of efficacy*
Gregorc 2018	II	≥2L	NGRhTNF + doxorubicin	TNF/CD13R	28	25%	3.2 mos	5.6 mos	Better results were documented in CT sensitive pts. *No further studies*
Morgensztern 2019	II	≥2L	RRx-001 -> CE	Myc	26	27%	7.5 mos	8.6 mos	
REPLATINUM Chiappori 2020	II	2L	RRx-001-> CE<br>CE rechallange	Myc	6<br>11	NR	7.1 mos<br>3.5 mos	8.2 mos<br>6.3 mos	Suspended during the pandemic

Owonikoko 2020	R II	2L	Alisertib (+ /- PTX)	AuroraKA	89<br>89	22%<br>18%	3.3 mos<br>2.1 mos	6.1 mos<br>5.4 mos	Among pts with CDK6, RBL1/2 alterations mPFS 3.6 vs 1.8 mos MS 7.2 vs 4.4 mos
Udagawa 2020	II	≥2L	gedatolisib	mTOR	12	0%	0.9 mos	5.8 mos	Selected pts whose SCLC harbouring mTOR pathway alterations
SUKSES Park 2020	II	≥2L	-adavosertib<br>- vistusertib<br>-AZD2811NP	WEE1<br>mTOR<br>AuroraKB	7<br>4<br>15	0%<br>0%<br>0%	1.3 mos<br>1.2 mos<br>1.2 mos	7.8 mos<br>11 mos<br>5.3 mos	
Carneiro 2023	Ib	≥2L	Mirzo-C (+/- PTX)	BCL-Xl	12	0%	NR	NR	*Lack of efficacy*
**Ongoing clinical trials**									
**Identifier**	**Phase**	**Setting**	**Drug (s)**	**Target**	**Pt N**	**Principal endpoint**	**Notes**		
NCT04560972	Ib	1L	CT +IO + LB100	Protein Phosphatase 2A	21	ORR	Recruiting USA		
NCT05745350	II	1L	Pembrolizumab + CT + Plinabulin	tubulin polymerization	45	12moPFS	Active, not yet recruiting		
NCT06030258	I/II	1L	CT+ Tislelizumab +IN1001	FAK	120	RD/PFS	Recruiting China		
PUMA-ALI-4201 NCT06095505	II	2L	Alisertib	Aurora Kinase A	60	ORR	Recruiting USA		
NCT04010357	II	2L	Abemaciclib	CDK4/6	29	ORR	Recruiting Last updated 2 Oct 2023 USA		
NCT05353439	I	2L	Tazemetostat + topotecan + pembrolizumab	EZH2	60	MTD	Recruiting USA		
NCT04699838	II	1L	CT+IO Durva + cerelasertib (ATR inhibitor)	ATR	30	PFS	Recruiting		
NCT05731518	I/II	2L	SC0245 (ATR inhibitor) + CPT11	ATR	67	RD/PFS	Recruiting		

Abbreviations: Ph = phase; Pt N = patient number; ORR = overall response rate; mPFS = median progression-free survival; MS = median survival; R II= randomized phase II trial; 2L = second-line; Alisertib = aurora kinase A inhibitor; PTX = paclitaxel; NGRhTNF = Asparagine–glycine–arginine–human tumour necrosis factor; BI2536 = PLK1, polo-like kinase 1, inhibitor; Linsitinib = IGF1R, insulin-like growth factor 1 receptor, inhibitor; ATR = ataxia telangiectasia and Rad3-related protein; Mirzo-C = Mirzotamab clezutoclax, a BCL-XL, B-cell lymphoma-extra large gene, inhibitor; gedatolisib = mTOR, mechanistic target of rapamycin, inhibitor; adavosertib = WEE-1 inhibitor, a G2 checkpoint cell cycle regulator; vistusertib = mTOR, inhibitor; AZD2811NP = an Aurora kinase B inhibitor; RRx-001= M2-M1 tumor associated macrophage repolarization and reversal of chemoresistance; CE = carboplatin/cisplatin etoposide; mos = months; CDK6 = cyclin-dependent kinase 6 gene; RBL1/2 = retinoblastoma-like 1/2 genes; CT = chemotherapy; RD=recommended dose; FAK=focal adhesion kinase; EZH2= Enhancer of Zeste Homologue2; ORR=overall response rate; RD=recommended dose; MTD= maximum tolerated dose; PFS= progression free survival.

Table S16: Ongoing clinical trials with ADC in ES-SCLC.

ID	Phase	Setting	N. Pts	Drug	Target	Primary Endpoint	Secondary Endpoints	Status and notes
NCT06227546	II	≥2L	17	Vobramitamab duocarmazine (MGC018)	B7-H3	ORR	PFS, OS	Active, recruiting
NCT06052423 ARTEMIS-007	II	1L	50	HS-20093	B7-H3	ORR	AEs, DCR, PFS, OS	Withdrawn
NCT04152499	I–II	≥2L	430*	SKB264	TROP2	MTD, ORR	DLTs, DOR, PFS, OS	Active, recruiting
NCT04826341	I–II	≥2L	85[&]	Sacituzumab govitecan + berzosertib	TROP2 + ATR inhibitor	MTD, ORR	PFS, OS, DOR	Recruiting.<br>Patients resistant to PARPi
NCT03221400	I–IIa	≥2L	340*	PEN-866	HSP90	DLT, ORR	DCR, PFS, OS, DOR	Recruiting
**NCT02187848**	I	≥2L	254*	Tusamitamab Ravtansine (SAR408701)	CEACAM5	MDT, ORR	PK, RP2D	Active, not recruiting

Legend: *Solid tumors (including SCLC). [&]included small cell lung cancer and extrapulmonary small cell neuroendocrine cancer. **Abbreviation:** ORR= overall response rate; PFS= progression free survival; OS= overall survival; AEs=adverse events; DCR=disease control rate; MDT= maximum dose tolerated; DLT=dose limiting toxicity; PARPi= Poly(ADP-ribose) polymerase inhibitors; HSP90=Heat shock protein 90; TRO2=trophoblast cell surface glycoprotein antigen 2; B7-H3=B7 homolog 3 protein; ATR=Ataxia telangiectasia mutated and Rad3 Related; CECAM5=Carcinoembryonic antigen-related cell adhesion molecule 5.

Table S17: Other ongoing trials in ES-SCLC [cancergov.trials]

Identifier	Phase	Pt N	Drugs	Setting	Principal Aim	Note
**Translational research**						
**MOSAIC NCT05933863**	R	200	ICI	1L	mPFS	immune microenvironment differences between subtypes to uncover ICI resistance mechanisms. Recruiting China
**Lung Cancer Registry NCT04654364**	IV	500	NA	NA	Observation	NSCLC/SCLC Recruiting Last updated 26 Aug 2022 Austria
**NCT05945745**	IV	500	NA	SCLC	Observation	Molecular analysis and treatment options Recruiting Shanghai
**NCT05066945**	IV	40	NA	1L	ctDNA	Recruiting China
**Vaccine**						
**NCT04397003**	II	27	Personalized vaccine + durvalumab	M	Safety	Recruiting USA
**VENEZO-LUNG NCT04487756**	Ib/II	20	Atezo + DC vaccine	M	PFS-rate 6mo	Recruiting Spain
**Acclaim3 NCT05703971**	Ib/II	62	quaratusugene ozeplasmid + atezo	M	MTD/PFS	Recruiting
**NCT05205421**	I	20	Oncolytic Virus Injection (RT-01)	≥2L	AEs/ORR	Recruiting Last updated 5 Aug 2022 China
**Miscellaneous**						
**NCT05874401**	IV	302	Topotecan +/- trilaciclib	2L	OS	Myelo-preservative Recruiting Europe
**NCT05299255**	II	40	Utiledone	3L	ORR	Epothilone similar to taxanes Recruiting China
**FASTimmune NCT05703997**	II	20	5-day diet restriction + atezo	M	6MO PFS rate	Not yet recruiting Italy
**NCT04919382**	II	56	Atezo + TMZ	2/3L	ORR	Recruiting Last updated 19 Dec 2023 USA
**RT/radiodrug**						
**NCT05595460**	Ib	31	RYZ101+ CT+IO	1L	RP2D	Radio-drug Recruiting USA
**NCT05142696**	Ib	39	[177Lu]Lu-DOTA-TATE+ CT + IO	1L	DLT	Recruiting USA/France/ Germany/Spain
**DARES NCT05068232**	II	49	Ablative RT + durvalumab	1L	PFS	Recruiting USA
**NCT06217757**	I/II	45	Olaparib + sugemalimab + CT + low dose RT	1L	RD	SLFN-11 Positive ES-SCLC Not yet recruiting China
**NCT06223711**	II	43	Chest RT + CT+ IO + SRT	1L	PFS	Oligometastatic up to 5 lesions

						Recruiting<br>Germany
**TRIPLEX**<br>**NCT05223647**	III	302	CT/IO+ chest RT<br>Vs<br>CT/IO	1L	1-yr OS	Recruiting<br>Northern Europe
**NCT06187740**	Ib	28	RT + IO	M	DLT	Recruiting<br>Shanghai
**CHESTRT**<br>**NCT05796089**	II	35	CHEST RT +<br>CT + IO	1L	Safety	recruiting<br>Australia
**NCT05765825**	II	61	Low dose chest RT + CT<br>+IO	1L	PFS	Recruiting<br>China
**NCT05403723**	Ib	50	CT + IO -> Chest RT for<br>pts SD/PD	1L	AEs	Recruiting<br>USA
**TESSERACT**<br>**NCT06110572**	I/II		CT/IO+ total body low<br>dose RT	1L	AEs	Recruiting<br>USA
**NCT06236997**	II	48	Adebrelimab (SHR1316)<br>+ CT +RT	1L	6mo PFSrate	Oligometastatic<br>Not yet recruiting<br>China
**PRIO trial**<br>**NCT04728230**	I/II	63	Olaparib + durva + CT	1L	DLT	Recruiting<br>USA
**NCT05484583**	II	58	Durva+CT<br>+ chest RT + SRT	M	OS	Oligometastatic<br>Not yet recruiting<br>China
**NCT06177925**	II	62	Adebrelimab (SHR1316)<br>+ CT +RT	1L	PFS	Oligometastatic<br>Recruiting<br>China
**NCT05544149**	II	42	Chest RT after CT +IO	M	Local<br>Recurrence free	Recruiting<br>China
**RAPTOR trial**<br>**NCT04402788**	II/III	138	Atezo +RT	M	PFS/OS	Recruiting<br>USA
**NCT05552846**	II	104	Consolidative RT after<br>CTIO	M	1-yr PFS rate	Recruiting<br>China
**NCT04923776**	II	18	RT on liver mets+ CT +IO	1L	PFS rate	Pts with liver mets<br>Recruiting<br>USA
**NCT06243003**	I/II	56	WBRT Hyppocampal<br>sparing + SRT	1L	DLT/1yr IC PFS	BM+<br>Recruiting<br>China
**PRIMALung**<br>**NCT04790253**	III	600	PCI<br>Vs<br>MRI surveillance	LD or ES	OS	After standard Tx<br>Recruiting<br>worldwide
**MAVERICK**<br>**SWOG S1827**<br>**NCT04155034**	III	668	PCI<br>Vs<br>MRI surveillance	LD or ES	OS	Recruiting<br>USA

Abbreviations: Ph = phase; Pt N = patient number; ORR = overall response rate; mPFS = median progression-free survival; mOS = median overall survival; R II= randomized phase II trial; L = line; CT = chemotherapy, RT = radiotherapy , IO = immunotherapy , MRI = magnetic resonance imaging, PCI = prophylactic cranial irradiation, MTD = maximum tolerated dose, DLT = dose limiting toxicity; ctDNA = circulating tumor DNA ; AE = adverse events ; TTD = therapeutic target database. Last accessed to clinicaltrial.gov on March 10, 2024.
